# Neuromuscular junction dysfunction in Lafora disease

**DOI:** 10.1242/dmm.050905

**Published:** 2024-10-14

**Authors:** Monica Shukla, Deepti Chugh, Subramaniam Ganesh

**Affiliations:** ^1^ Department of Biological Sciences and Bioengineering, Indian Institute of Technology, Kanpur 208016, India; ^2^ Mehta Family Centre for Engineering in Medicine, Indian Institute of Technology, Kanpur 208016, India; ^3^ Gangwal School of Medical Sciences and Technology, Indian Institute of Technology, Kanpur 208016, India

**Keywords:** Neurodegenerative disorder, Glycogen storage disease, Progressive myoclonus epilepsy, Autophagy defect, Metabolic disorders

## Abstract

Lafora disease (LD), a fatal neurodegenerative disorder, is caused by mutations in the *EPM2A* gene encoding laforin phosphatase or *NHLRC1* gene encoding malin ubiquitin ligase. LD symptoms include epileptic seizures, ataxia, dementia and cognitive decline. Studies on LD have primarily concentrated on the pathophysiology in the brain. A few studies have reported motor symptoms, muscle weakness and muscle atrophy. Intriguingly, skeletal muscles are known to accumulate Lafora polyglucosan bodies. Using laforin-deficient mice, an established model for LD, we demonstrate that LD pathology correlated with structural and functional impairments in the neuromuscular junction (NMJ). Specifically, we found impairment in NMJ transmission, which coincided with altered expression of NMJ-associated genes and reduced motor endplate area, fragmented junctions and loss of fully innervated junctions at the NMJ. We also observed a reduction in alpha-motor neurons in the lumbar spinal cord, with significant presynaptic morphological alterations. Disorganised myofibrillar patterns, slight z-line streaming and muscle atrophy were also evident in LD animals. In summary, our study offers insight into the neuropathic and myopathic alterations leading to motor deficits in LD.

## Teaser

**Summary:** Motor symptoms, muscle weakness and muscle atrophy have been reported in patients with Lafora disease. Here, using a mouse model, we demonstrate that motor deficits in Lafora disease are caused by neuropathic and myopathic alterations.

## INTRODUCTION

Lafora disease (LD) [Online Mendelian Inheritance in Man (OMIM) 254780] is a rare neurodegenerative disorder that typically manifests in adulthood. It is inherited in an autosomal-recessive manner and is known to cause severe and debilitating symptoms such as epileptic seizures, myoclonus, ataxia and cognitive decline (Upadhyay et al., 2017; Mitra et al., 2022; Parihar and Ganesh, 2024; Zimmern and Minassian, 2024). LD is caused by genetic mutations in *EPM2A* or *NHLRC1*, which encode laforin and malin proteins, respectively, and functional loss of one of these two proteins results in dysregulation of glycogen metabolism, leading to the formation of abnormal glycogen aggregates known as Lafora bodies (LBs) in different tissues, including the brain, muscle and liver (Parihar et al., 2018; Mitra et al., 2022; Parihar and Ganesh, 2024; Minassian, 2001; Zimmern and Minassian, 2024). Besides glycogen metabolism (Worby et al., 2006; Tagliabracci et al., 2007, 2008; Singh et al., 2012; Duran et al., 2014), *EPM2A* or *NLHRC1* gene defects are known to affect diverse cellular pathways, including autophagy, the ubiquitin–proteasome system (UPS), neuroinflammation and endoplasmic reticulum stress (Parihar and Ganesh, 2024). Several studies have extensively investigated alterations of these pathways in LD mouse models (Garyali et al., 2009; Vernia et al., 2009; Aguado et al., 2010; Puri and Ganesh, 2010; Criado et al., 2012; Puri et al., 2012; Zeng et al., 2012; Sharma et al., 2013; Singh et al., 2013; Garyali et al., 2014; Sinha et al., 2021a,b). Most studies on LD have primarily concentrated on the pathophysiology and clinical manifestations within the central nervous system (CNS). The emphasis on this area is motivated by the obvious and significant decline in neurological function and cognitive abilities observed in individuals affected by LD (Singh and Ganesh, 2009). Nevertheless, several case studies indicate that individuals with LD exhibit motor symptoms that are characterised by muscle weakness and muscle atrophy (Schwarz and Yanoff, 1965; Coleman et al., 1974; Neville et al., 1974; Lee et al., 2008; Rudenskaia et al., 2010; Al Mufargi et al., 2020; d'Orsi et al., 2022; Zeka et al., 2022). The utilisation of mouse models for LD – specifically, the laforin-deficient mice – has proven to be an effective means of replicating the various symptoms and pathological characteristics associated with LD, as observed in human patients (Ganesh et al., 2000, 2002; DePaoli-Roach et al., 2010; Turnbull et al., 2010; Valles-Ortega et al., 2011; Criado et al., 2012; Brewer and Gentry, 2019). LBs can be detected in both the brain and peripheral tissues of murine models beginning at 2 months of age. The deposition of LBs is followed by cognitive deficits and epileptic seizures (Ganesh et al., 2002). In addition, LD mouse models exhibit muscle weakness and impaired motor coordination, which closely resemble the symptoms observed in LD patients (Ganesh et al., 2002, 2006; d'Orsi et al., 2022). However, the specific neurobiological mechanisms responsible for these impairments are still not well understood. Therefore, it is necessary to conduct research that specifically aims to comprehend these peripheral manifestations, specifically the role of skeletal muscles that exhibit significant accumulation of LBs (Coleman et al., 1974; Neville et al., 1974; Turnbull et al., 2011, 2016; Parihar et al., 2018). Towards this direction, the current study utilising an established LD mouse model (laforin-deficient and malin-deficient mice) demonstrates that LD pathology is strongly linked to structural abnormalities and impaired neuromuscular junction (NMJ) functionality. The dysfunction observed could be attributed to either a primary defect in the NMJ caused by loss-of-function mutations in the *Epm2a* or *Nhlrc1* gene or a secondary result of impairments in other interconnected functional pathways.

## RESULTS

### Electrophysiological assessment of NMJ transmission defects in the LD mouse model

**Fig. 1.**
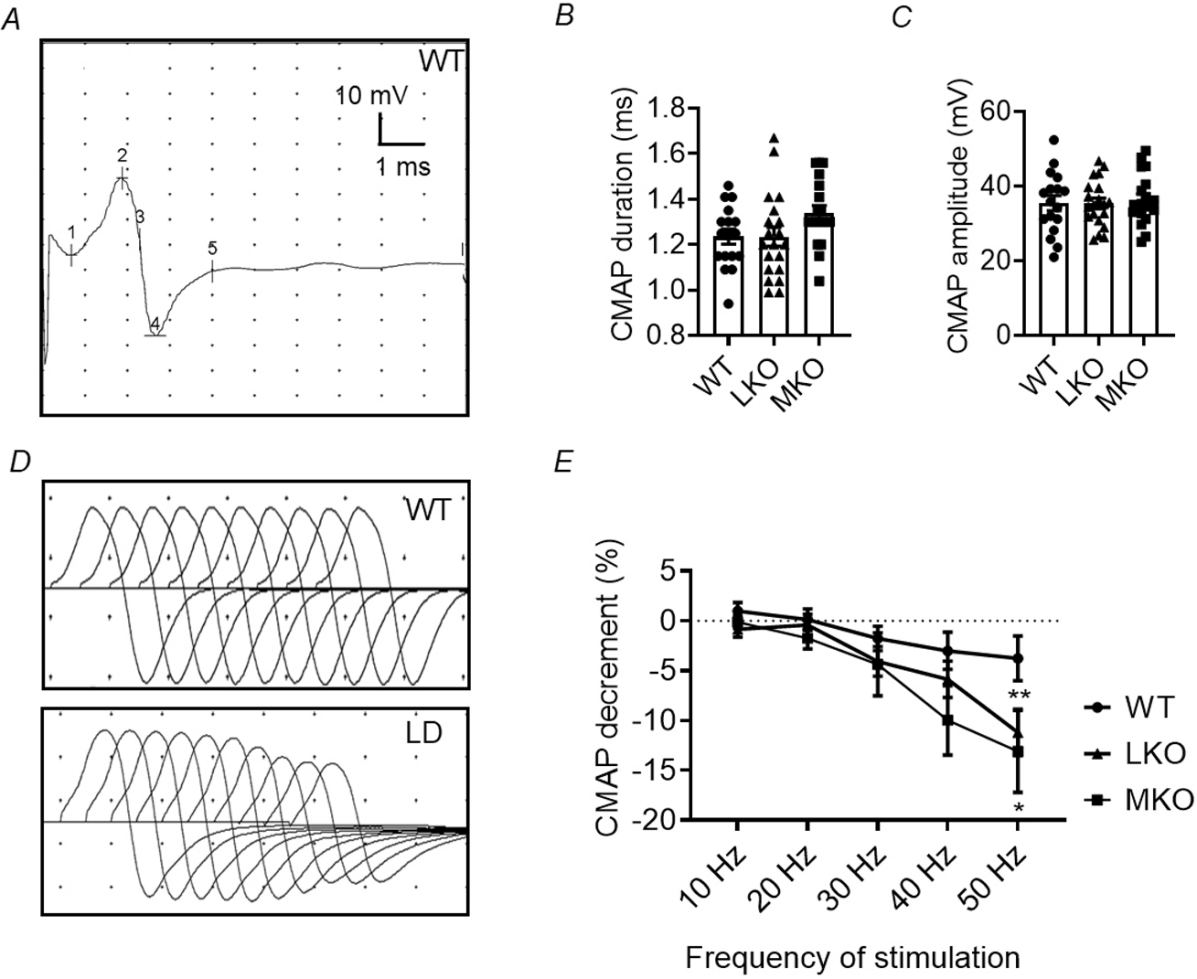
***In vivo* electrophysiological assessment of neuromuscular junction (NMJ) transmission in the Lafora disease (LD) mouse model.** (A) Representative traces of compound muscle action potential (CMAP) recordings from the gastrocnemius muscle in a wild-type (WT) animal. Numbers indicate onset (1), negative peak (2), baseline crossing (3), positive peak (4) and end (5) of the CMAP. (B,C) CMAP duration (ms; B) and CMAP amplitude (mV; C) of 10-month-old WT, laforin-deficient (LKO) and malin-deficient (MKO) mice. Each bar represents the mean±s.e.m. (*n*=17 WT, *n*=20 LKO and *n*=18 MKO; one-way ANOVA with Sidak's multiple comparison test). No significant differences were observed for CMAP duration (WT, 1.23±0.03 ms; LKO, 1.23±0.04 ms; MKO, 1.34±0.03 ms; *P*=0.07) and CMAP amplitude (WT, 35.49±2.01 mV; LKO, 35.42±1.39 mV; MKO, 36.45±1.63 mV; *P*=0.88). (D) Representation of ten consecutive CMAP discharges resulting from repetitive nerve stimulation (RNS) in WT (top) and LKO (bottom) mice. Note the CMAP decremental responses with an increase in stimulation frequency in LD animals. The first CMAP amplitude stayed the same at all stimulation frequencies (from 10 Hz to 50 Hz); decrement was noted in the tenth response in LD animals. (E) Percentage CMAP decrement following RNS in 10-month-old LKO and MKO mice compared to WT mice. Note the significantly increased CMAP decrement at 50 Hz frequency of stimulation in the LKO and MKO mice at 10 months (*n*=17 WT, *n*=20 LKO and *n*=18 MKO; two-way ANOVA with Sidak's multiple comparison test; *P<0.05, **P<0.01).

The utilisation of electrophysiological recording techniques – specifically, compound muscle action potential (CMAP) and repetitive nerve stimulation (RNS) – enables the evaluation of neuromuscular function in a manner that is minimally invasive. The CMAP response is a measure of the combined depolarisation of muscle fibres in a muscle following supramaximal nerve stimulation. In the current investigation, we conducted measurements of CMAP and RNS on the gastrocnemius muscle of mice subsequent to stimulation of the sciatic nerve (Fig. 1A,D). There were no significant differences observed in the duration of CMAP between wild-type (WT), laforin-deficient (LKO) and malin-deficient (MKO) mice at 10 months (Fig. 1B). Likewise, there were no significant differences observed in the amplitude of CMAP between the WT, LKO and MKO mice at 10 months of age (Fig. 1C). Similar observations were made in the 5-month-old LKO mice compared to age-matched WT control mice (Fig. S1A,B). Furthermore, the LD mouse models, including both LKO and MKO mice, were subjected to an RNS assessment, which is considered a diagnostic benchmark for individuals with NMJ disorders. To evaluate the impairments in NMJ transmission, the decrement in CMAP amplitude was measured. This measurement was taken in response to RNS at different frequencies. The presence of defects in NMJ transmission was determined if the amplitude decreased by at least 10% after ten repetitive stimuli, as shown in Fig. 1D. A notable reduction in CMAP response was observed in 10-month-old LKO and MKO mice, particularly at higher frequencies of stimulation. The CMAP response in LKO and MKO mice showed a decrement of 11.21±2.33% and 11.99±4.05%, respectively, at 50 Hz, significantly different from the response in age-matched WT controls (3.75±2.24%) (Fig. 1E). It is worth noting that the CMAP decrement was also observed in the younger LKO cohort (14.93±4.26%) at 50 Hz at the age of 5 months (Fig. S1C). We noted the presence of these impairments in NMJ transmission efficiency in ∼50-60% of the LKO and MKO mice at 10 months. Interestingly, LD animals showed NMJ transmission dysfunction from the age of 5 months, as shown in Fig. S2A,B. Taken together, these findings confirm impaired NMJ performance in the hindlimb skeletal muscle of the LD mouse model.

### NMJ morphological alterations and innervation pattern in gastrocnemius muscle of laforin-deficient mice

**Fig. 2.**
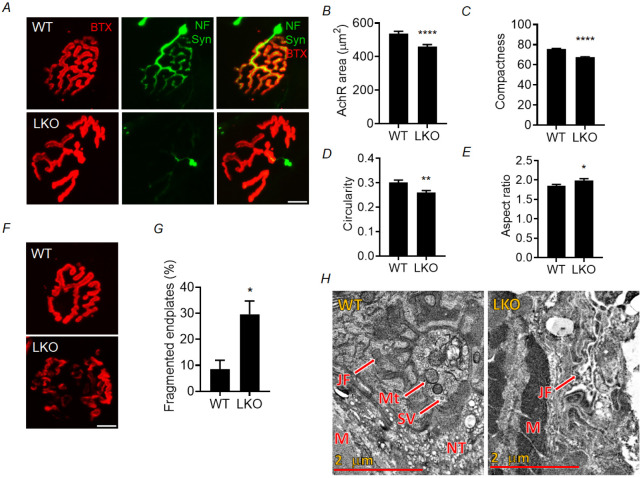
**Altered NMJ morphology in gastrocnemius muscle of the LD mouse model.** (A) Representative immunofluorescence images showing NMJ morphology in 10-month-old WT and LKO animals, in which postsynaptic acetylcholine receptors (AChRs) are stained with Alexa Fluor 594-conjugated α-bungarotoxin (BTX; red), presynaptic nerve is stained with anti-neurofilament (NF) antibody (green), and presynaptic nerve terminals are stained with anti-synapsin-1 (Syn) antibody (green). Note the highly compact pretzel-shaped NMJ structure in WT animals. In contrast, dispersed and less complex NMJ morphology is observed in LKO mice. (B-E) Quantification of NMJ morphological features, indicating a significant reduction in postsynaptic AchR area (B), compactness (C) and circularity (D), and an increase in aspect ratio (E) in 10-month-old LKO animals compared to WT animals. (F) Representative immunofluorescence images showing a fragmented NMJ in LKO mice, as opposed to an intact junction in WT mice, at 10 months of age. (G) Quantification of the relative percentage of fragmented endplates in 10-month-old LKO animals compared to age-matched WT animals. (H) Representative transmission electron microscopy (TEM) images showing the neuromuscular junction in 10-month-old WT and LKO animals. Note the well-defined postsynaptic junctional folding (JF; marked by arrow) and nerve terminal (NT; marked by arrow) filled with an abundant number of synaptic vesicles (SV; marked by arrow) at the presynaptic region in WT animals. In contrast, fewer postsynaptic junctional foldings are present in LKO animals (JF; marked by arrow). Moreover, such junctional foldings were discontinuous in some places. M, muscle; Mt, mitochondria. For B-E and G, each bar represents the mean±s.e.m.; 70-100 NMJs were analysed per animal, and three animals were used for each genotype (WT and LKO) (unpaired two-tailed Student's *t-*test; *P<0.05, **P<0.01, **** *P*<0.0001). Scale bars: 10 µm (A,F) and 2 µm (H).

Given that we observed similar changes in the electrophysiological properties of the NMJ in the LKO and MKO mice, we further proceeded with the LKO animals only for the subsequent set of experiments. The observed electrophysiological changes could be attributed to alterations in the structure of both the presynaptic and postsynaptic regions in LKO mice. In order to evaluate potential alterations in the structure of the NMJ, we conducted immunofluorescence experiments in WT and LKO animals. Frozen tissue sections of the gastrocnemius skeletal muscle were subjected to triple immunolabeling. An anti-neurofilament antibody was used to stain the axons of motor neurons selectively, and an anti-synapsin antibody was used to identify the presynaptic nerve terminals. Additionally, α-bungarotoxin (BTX) was used to stain the postsynaptic acetylcholine receptors (AchRs) that constitute the motor endplate. Confocal microscopy was carried out to acquire fluorescent images. As shown in Fig. 2A, in the 10-month-old WT mice, a clearly structured NMJ morphology was observed, both at the presynaptic and postsynaptic locations. The junctions exhibited a dense and intricate arrangement, characterised by a distinctive pretzel-like shape and notable abundance of postsynaptic folding. In contrast, the NMJs of 10-month-old LKO animals exhibited various morphological changes at the site of a junction, as shown in Fig. 2A. Our results demonstrate a significant decrease in the AchR area, compactness and circularity in LKO mice compared to age-matched WT mice (Fig. 2B-D; Table S1). The aspect ratio, however, showed a significant increase in LKO mice compared to WT mice (Fig. 2E; Table S1). We also analysed the postsynaptic motor endplate fragmentation (Fig. 2F). The fragmentation of a specific junction was determined by the presence of five or more irregular clusters at the postsynaptic site. There was a significant increase in the occurrence of fragmented endplates in the 10-month-old LKO mice compared to WT mice (Fig. 2G; Table S1). Comparable morphological modifications were also observed in the 5-month-old LKO animals (Fig. S3A-G, Table S2). To gain more insight into the morphological changes, we employed transmission electron microscopy (TEM) to determine the ultrastructure of the NMJ in the gastrocnemius muscle of LD animals. As shown in Fig. 2H, the WT animals exhibited an NMJ morphology that was well preserved, characterised by the presence of properly formed infoldings that created junctional folds at the postsynaptic region. In contrast, LKO animals exhibited invaginations at the postsynaptic region that were less intricate, indicating a comparatively less complex morphology of the NMJ. Taken together, our study results indicate that LKO mice display significant alterations in NMJ morphology at both the 10-month and 5-month time points.

**Fig. 3.**
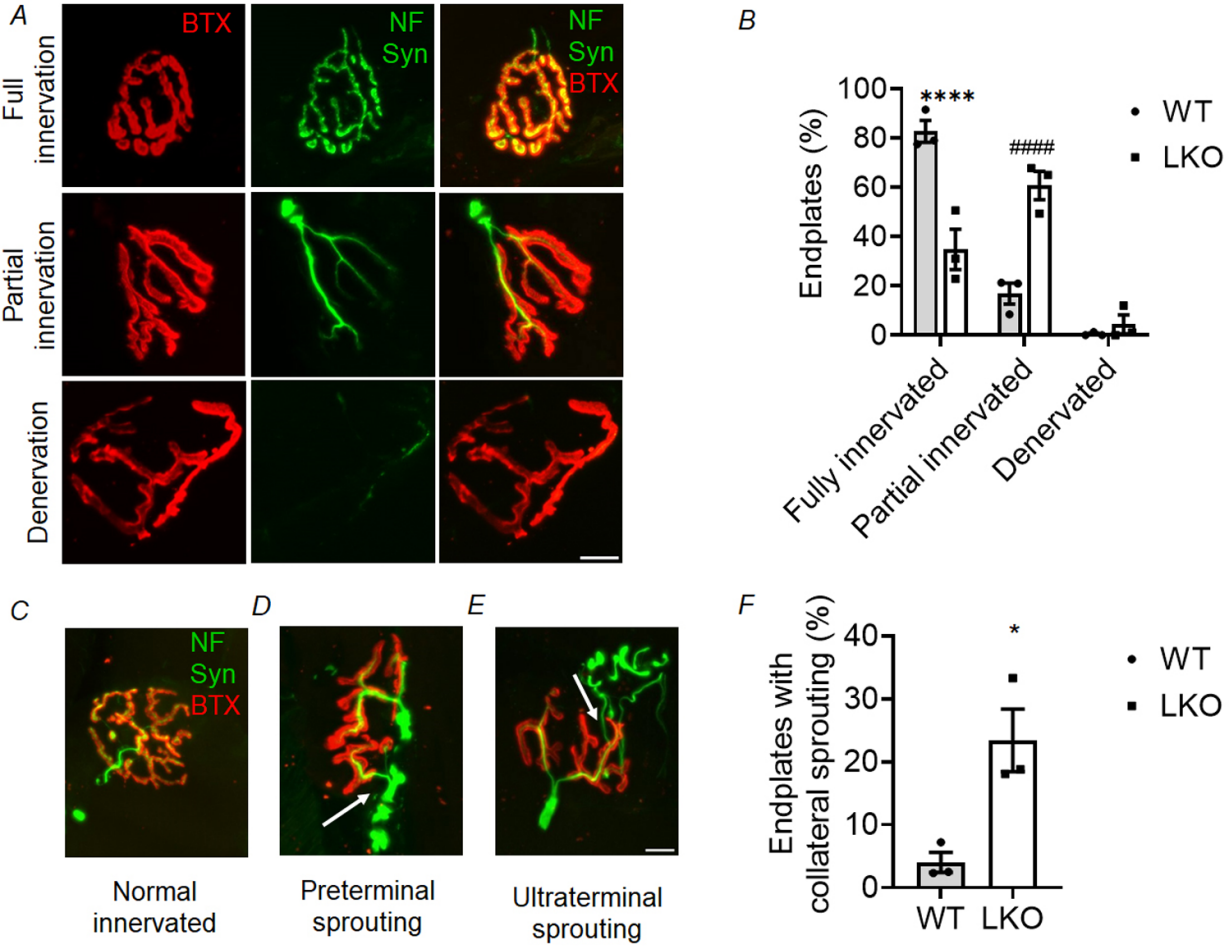
**NMJ innervation pattern and nerve collateral sprouting in gastrocnemius muscle of the LD mouse model.** (A) Representative immunofluorescence images showing the NMJ innervation pattern categorised as full**,** partial and denervated junctions. Sections were stained with BTX (red) and a combination of anti-neurofilament (NF; green) and anti-synapsin-1 (Syn; green) as detailed in Fig. 2A. (B) Quantification of the relative percentage of endplates exhibiting full, partial and denervated junctions in 10-month-old LKO animals compared to age-matched WT control animals. Each bar represents the mean±s.e.m.; 70-100 NMJs were analysed per animal, and three animals were used for each genotype (WT and LKO) (two-way ANOVA with Sidak's multiple comparison test; ****P<0.0001). (C-E) Immunofluorescence images representing the phenomenon of collateral sprouting at NMJs. (C) In normal innervated NMJ, the presynaptic axon terminal innervates only one motor endplate without collateral sprouting. (D) In the case of preterminal sprouting, the motor endplate is innervated by a newly formed thin axonal branch originating from an area immediately proximal to the NMJ (white arrow). (E) In the case of ultra-terminal sprouting, new axonal sprouts emanate from but grow beyond the endplate region to innervate the nearby denervated junction (white arrow). (F) Relative percentage of endplates with collateral sprouting in 10-month-old LKO animals compared to age-matched WT control animals. Note the increased frequency of collateral sprouting in the 10-month age group compared with WT control animals. Each bar represents the mean±s.e.m.; 70-100 NMJs were analysed per animal, and three animals were used for each genotype (WT and LKO) (unpaired two-tailed Student's *t-*test; *P<0.05). Scale bars: 10 µm.

We next assessed the innervation status of the NMJ in order to gain further understanding of its functionality in the LKO mouse model. The nerve innervation pattern within the motor endplate was examined by analysing the overlapping area positive for synapsin (a presynaptic protein) and the BTX-labelled postsynaptic AchRs, as depicted in Fig. 3A. Analysis of the stained NMJs indicated that a significant proportion of the motor endplates in the 10-month-old WT mice was completely innervated (Fig. 3A,C). A smaller proportion of junctions exhibited partial innervation, while the presence of denervated junctions was nearly absent. In contrast, the LKO mice demonstrated a significant decrease in the relative percentage of fully innervated junctions and a significant increase in the number of partially innervated junctions. A minority of denervated junctions was also detected in the LKO mice (Fig. 3B; Table S3). The 5-month-old LKO mice exhibited similar alterations (Fig. S4A,B, Table S4). However, it is worth noting here that the extent of loss of fully innervated junctions was less pronounced in 5-month-old than in 10-month-old LKO mice. Collectively, our study provides evidence for the progressive deterioration of NMJ architecture and innervation in the skeletal muscle of the LKO mice.

Collateral sprouting is an inherent regenerative mechanism that promotes the restoration of function in a pathological state. This phenomenon encompasses the process by which intact neurons generate new axonal connections, allowing them to establish innervation with adjacent denervated endplates (Rosenheimer, 1990a; Marshall and Farah, 2021). The lack of significant alterations in the compound muscle action potential (CMAP) response of the gastrocnemius muscle prompted us to investigate collateral sprouting as a potential compensatory regenerative process that may occur in LKO mice. In this study, we present empirical evidence of collateral sprouting in 10-month-old LKO animals based on our immunostaining observations. We identified two distinct patterns of collateral sprouting based on the source of new axonal projections. The first pattern, known as preterminal sprouting, involves the generation of new axonal projections from an area located directly adjacent to the NMJ (see Fig. 3D). The second pattern, referred to as ultra-terminal sprouting, involves the emergence of new axonal sprouts from the endplate region that extends beyond it to innervate nearby denervated junctions, either on their own or on neighbouring muscle fibres (see Fig. 3E). This observation demonstrates the regenerative potential of the NMJ and the capacity of undamaged neurons to form novel connections after denervation in the LKO mouse model. The analysis of sprouting patterns using a semi-quantitative approach demonstrated a significant elevation in the extent of collateral sprouting in 10-month-old LKO mice compared to age-matched WT control mice (Fig. 3F). A similar trend was observed in the 5-month-old LKO mice, although the difference was not statistically significant (Fig. S4C,D). This observation indicates that younger LKO mice initially display a low proportion of endplates with collateral sprouting, which gradually increases over time. These findings indicate that there is a positive correlation between age and the occurrence of compensatory regenerative collateral sprouting.

### Changes in the expression levels of genes implicated in neuromuscular functions in laforin-deficient mice

In order to gain a deeper understanding of the molecular processes that contribute to the dysfunction of the NMJ, we utilised quantitative reverse transcription polymerase chain reaction (qRT-PCR) to examine the expression levels of specific genes involved in this process. These genes include *Agrn*, *Musk*, *Lrp4*, *Dok7* and *Rapsn*, which are known to play a critical role in the clustering of postsynaptic AchRs and offer valuable insights into the intricate molecular interactions occurring at the NMJ (Zong and Jin, 2013). A significant reduction was noted in the relative expression levels of *Agrn* and *Musk* genes in 10-month-old LKO animals compared to WT animals (Fig. S5A). In the younger age groups, a comparable pattern was observed for all genes, with *Lrp4* showing a significant difference (Fig. S5B). Additionally, we assessed the mRNA expression level of acetylcholine esterase (*Ache*), a regulator of acetylcholine levels at the synapse. However, no significant differences in *Ache* levels were observed between WT and LKO animals at the designated time points (Fig. S5A,B). Collectively, our findings provide evidence that the modification of postsynaptic signalling at the NMJ may play a role in the deterioration of NMJ structure and function observed in the LKO mouse model.

### Loss of alpha-motor neurons in the ventral horn of the spinal cord in laforin-deficient mice

**Fig. 4.**
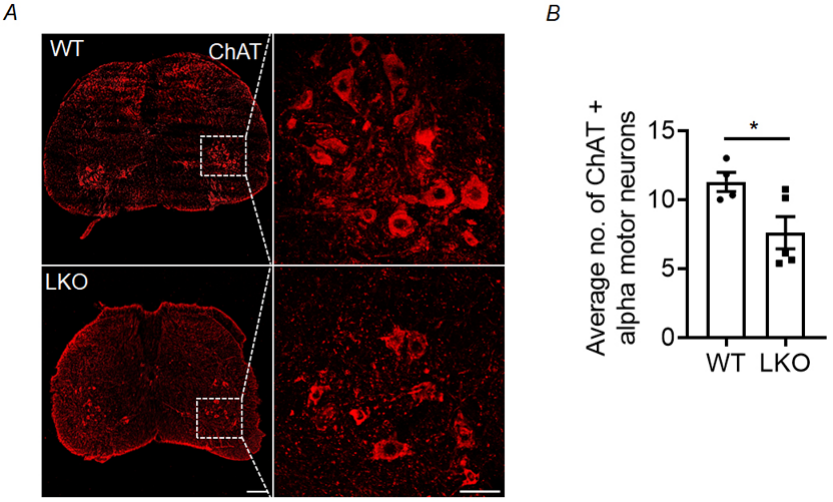
**Loss of ventral horn alpha-motor neurons in the lumbar region of the spinal cord in the LD mouse model.** (A) Representative images of choline acetyltransferase (ChAT) staining showing alpha-motor neurons in the ventral horn region of the lumbar spinal cord in WT and LKO animals. (B) Quantification of the average number of ChAT*-*positive alpha-motor neurons per side of the lumbar spinal cord in the ventral horn region in 10-month-old LKO animals compared to their age-matched WT littermates. Each bar represents the mean±s.e.m. [*n*=4-5 animals for each genotype (WT and LKO); unpaired two-tailed Student's *t-*tests; *P<0.05]. Scale bars: 200 µm (left) and 50 µm (right).

Age-dependent neurodegenerative changes have been well characterised in LD models. Nevertheless, investigations on motor neurons in the spinal cord, which offer direct innervation to the skeletal muscle, have not been conducted thus far. Thus, we quantified the number of choline acetyltransferase (ChAT)-positive alpha-motor neurons in the ventral horn of the spinal cord that specifically innervate hindlimb skeletal muscle. For this, we carried out immunofluorescence microscopy to detect motor neurons with the marker protein ChAT (Fig. 4A). As shown in Fig. 4B, there was a significant reduction in the number of ChAT-positive cells in the spinal cord in 10-month-old LKO mice compared to age-matched WT animals, suggesting the loss of alpha-motor neurons in the ventral horn in LKO animals. Similar observations were made in the 5-month-old animals, indicating the initiation of degenerative alterations prior to reaching 5 months of age (Fig. S6A,B).

### LB inclusions and increased neuroinflammation in the spinal cord of laforin-deficient mice

**Fig. 5.**
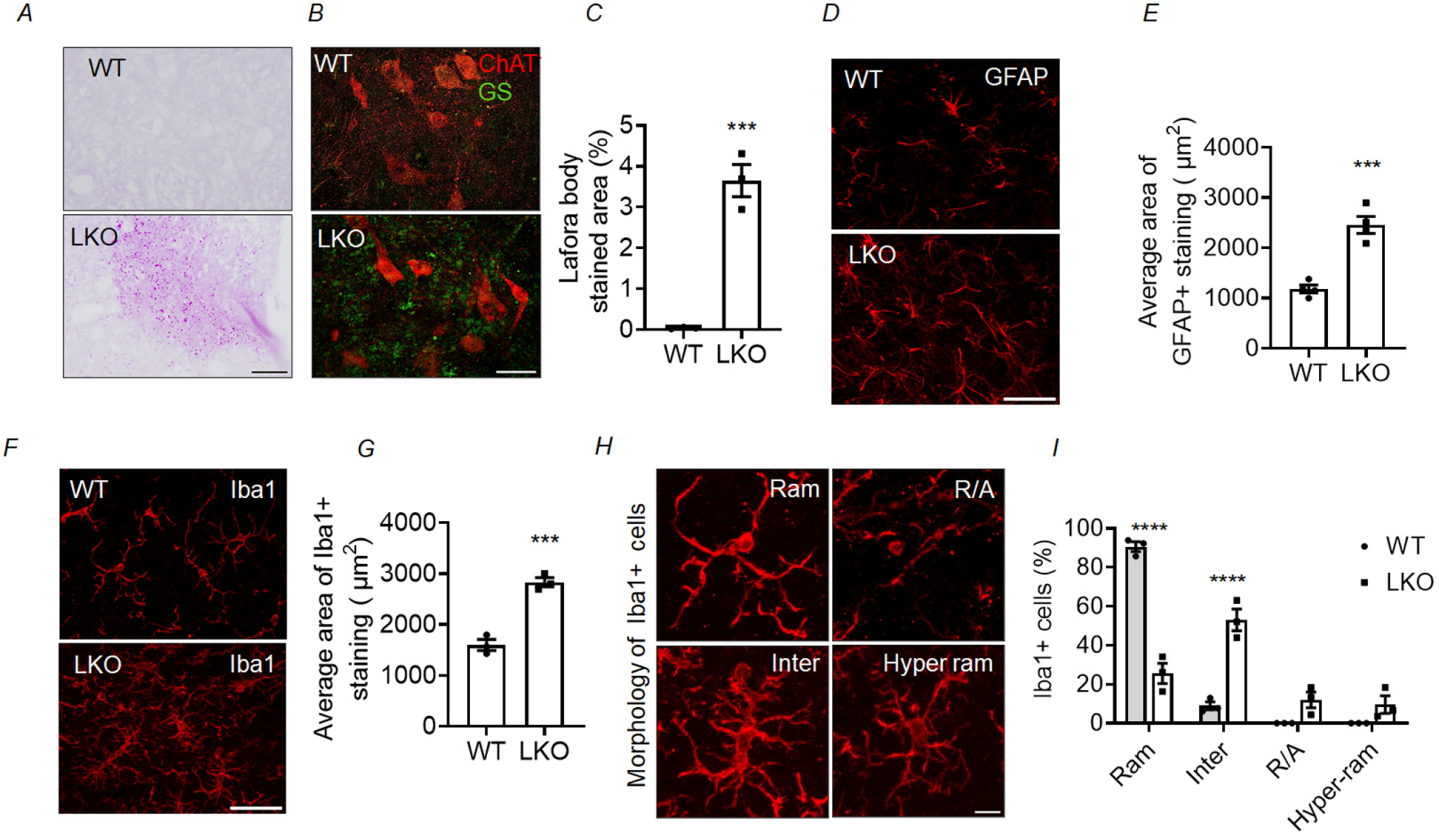
**Accumulation of Lafora bodies (LBs) and increased gliosis in the lumbar region of the spinal cord in the LD mouse model. (**A) Representative images showing periodic acid-Schiff (PAS)-positive LB accumulation in the ventral horn region of the spinal cord in LKO animals. (B) Representative images showing anti-glycogen synthase (GS) and anti-ChAT co-staining in the spinal cord of LKO animals. Note the GS-positive LBs (green) in the sections of LKO animals. (C) Percentage of LB-positive stained area in the ventral horn region of the spinal cord in 10-month-old LKO animals. Each bar represents the mean±s.e.m. [*n*=3 animals for each genotype (WT and LKO); unpaired two-tailed Student's *t-*test; ***P<0.001]. (D) Representative images from spinal cord sections showing the distribution of GFAP^+^ astrocytes in the ventral horn region of WT and LKO animals. (E) Average area occupied by the GFAP^+^ cells in 10-month-old LKO animals and age-matched WT animals. Each bar represents the mean±s.e.m. [*n*=4 animals for each genotype (WT and LKO); unpaired two-tailed Student's *t-*test; ***P<0.001]. (F) Representative images from spinal cord sections showing the distribution of Iba1^+^ microglial cells in the ventral horn region of WT and LKO animals. (G) Average area occupied by the Iba1^+^ cells in 10-month-old LKO animals and age-matched WT animals. Each bar represents the mean±s.e.m. [*n*=3 animals for each genotype (WT and LKO); unpaired two-tailed Student's *t-*test; ***P<0.001]. (H) Representative images showing the different morphological phenotypes of Iba1^+^ cells: ramified cell possessing small cell body and highly branched thin processes (Ram), intermediate cell with elongated cell body and thickened and shorter cell processes (Inter), round/amoeboid cell with round and swollen cell body with no processes (R/A) and hyper-ramified cell with processes having bushy-like appearance (Hyper ram). (I) Percentages of microglial cells exhibiting the four different morphological phenotypes in LKO animals and WT control animals. Each bar represents the mean±s.e.m.; two-way ANOVA with Sidak's multiple comparison test [*n*=3 animals for each genotype (WT and LKO); ****P<0.0001]. Scale bars: 50 µm (A,D,F) and 10 µm (B,H).

To gain a deeper understanding of the underlying mechanisms of loss of alpha-motor neurons in the LKO mice, we evaluated the presence of LB inclusions and neuroinflammation in the spinal cord. LBs are accumulations of insoluble, hyperphosphorylated polyglucosans composed of poorly branched glycogen molecules (Anraku and Kawasaki, 1966; Namba and Ota, 1966; Yokoi et al., 1968; Sullivan et al., 2017; Brewer et al., 2020). Periodic acid–Schiff (PAS)-positive LB inclusions were observed in the spinal cord of the LKO mice (Fig. 5A). Significantly, within the ventral horn of the spinal cord, a region in which degeneration of motor neurons was detected, ∼2-4% of the area exhibited distinct PAS-positive staining in the LKO mice at both 10 months (Fig. 5A,C) and 5 months (Fig. S7A,B) of age. Because glycogen synthase (GS) is known to colocalise with LBs in LD models (Varea et al., 2021), we carried out immunofluorescence staining to show the presence of LBs in the ventral horn region of the spinal cord. Indeed, the GS-positive LBs were detected in proximity to motor neurons in the spinal cord (Fig. 5B). Next, to assess for neuroinflammatory response in LD mice, we evaluated the expression of two established markers: glial fibrillary acidic protein (GFAP), for labelling astrocytes, and ionised calcium-binding adaptor molecule 1 (Iba1), for labelling microglial cells in the ventral horn of the spinal cord. The immunofluorescence analyses demonstrated a statistically significant increase in the expression of GFAP and Iba1 in 10-month-old LKO mice compared to age-matched WT control mice (Fig. 5D-G). Both proteins also showed a similar increase in the 5-month-old LKO animals (Fig. S7C-F). Microglial cells are recognised for displaying distinct morphological forms that can be classified into four phenotypes based on their level of activation: ramified (Ram), intermediate (Inter), round/amoeboid (R/A) and hyper-ramified (Hyper ram) (Fig. 5H). Therefore, we wanted to ascertain whether their relative proportion is altered in the LKO animals and conducted a quantitative analysis of the morphology of microglial cells. Our observations revealed a significant decrease in the proportion of microglial cells with normal, healthy ramified morphology and a significant increase in the proportion of microglial cells with activated intermediate, round/amoeboid and hyper-ramified morphology in the LKO animals at both 10 and 5 months of age (Fig. 5I; Fig. S7G). We provide evidence that motor neurons labelled with ChAT in the ventral horn of the spinal cord overlapped with regions exhibiting activated astroglia and microglia (Fig. S8A,B). These findings suggest that gliosis may play a causal role in the observed depletion of alpha-motor neurons in the LKO mice.

### Altered sciatic nerve morphology in laforin-deficient mice

**Fig. 6.**
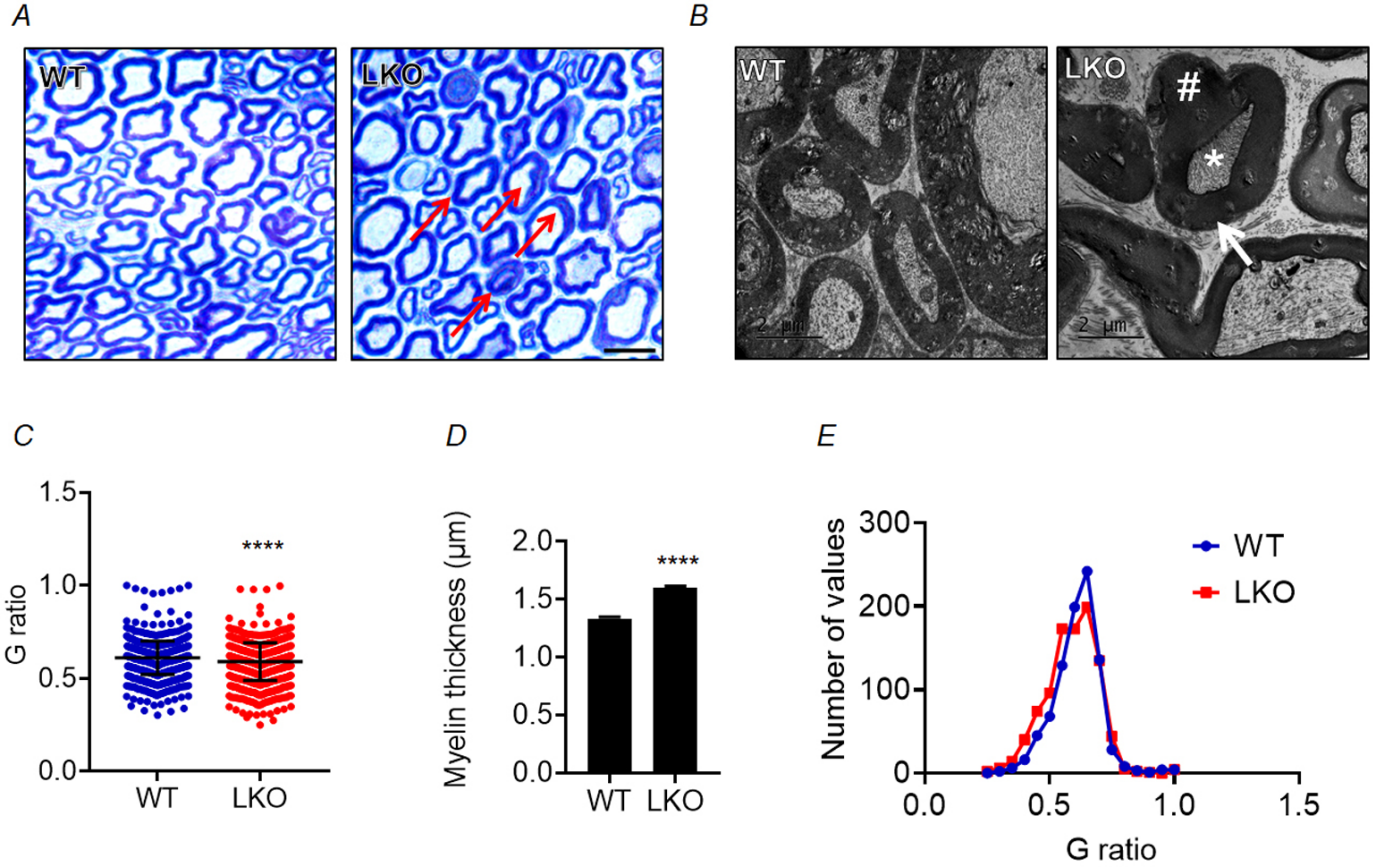
**Sciatic nerve morphology and myelination status are altered in the LD mouse model.** (A,B) Representative images of semi-thin (A) and ultra-thin (B) transverse sections of the sciatic nerve depicting nerve fibre morphology in 10-month-old WT and LKO mice. Note the thick myelin sheath in the axons of LKO animals (indicated by red arrows in A and white arrow in B). In B, ‘*’ marks the axon, and ‘#’ marks the myelin sheath. (C-E) Charts showing G ratio, myelin thickness, and relative distribution of G ratios across all myelinated nerve fires in 10-month-old LKO animals compared to WT animals. Axons of LKO animals show less G ratio (C) compared with that of WT animals, indicating more myelination (D) in the axons of LKO animals than in WT animals. In C, each bar represents the mean±s.d.; in D, each bar represents the mean±s.e.m. 270-300 nerve fibres were analysed from each animal, and three animals were used for each genotype (WT and LKO) (unpaired two-tailed Student's *t-*test; ****P<0.0001). Scale bars: 10 µm (A) and 2 µm (B).

The axon bundles of ventral horn alpha-motor neurons comprising sciatic nerve directly innervate the hindlimb skeletal muscle. To evaluate the morphology of peripheral nerves and the status of myelination in the LKO mouse model, the sciatic nerves were extracted from both 10- and 5-month-old animals. At each time point, three WT and three LKO animals were analyzed. Semi-thin cross-sections of these nerves were then stained with Toluidine Blue for assessment purposes (Fig. 6A). The G ratio, which quantifies the ratio between the diameter of axons and the diameter of fibres, was assessed to investigate the relationship between axonal myelination and axon diameter. Importantly, there was a significant decrease in the G ratio in 10-month-old LKO mice compared to age-matched WT mice (Fig. 6C). In addition, myelin thickness was determined through the subtraction of the axonal diameter from the combined diameter of the fibre, which encompasses both the axon and the myelin sheath. There was a significant increase in the thickness of myelin in the axons of the sciatic nerve in 10-month-old LKO mice compared to age-matched WT control mice (Fig. 6D). Similar observations were made in the 5-month-old LKO mice as well, both for the G ratio and for myelin thickness (Fig. S9A-C). These observations exhibited consistency among all axons, as shown in Fig. 6E and Fig. S9D. In addition, a qualitative assessment was conducted on ultra-thin sections of the sciatic nerve using transmission electron microscopy (TEM). This analysis revealed atypical morphological characteristics, specifically an observed increase in myelination within the nerve fibres of LKO mice (Fig. 6B).

### Muscle pathology in laforin-deficient mice

**Fig. 7.**
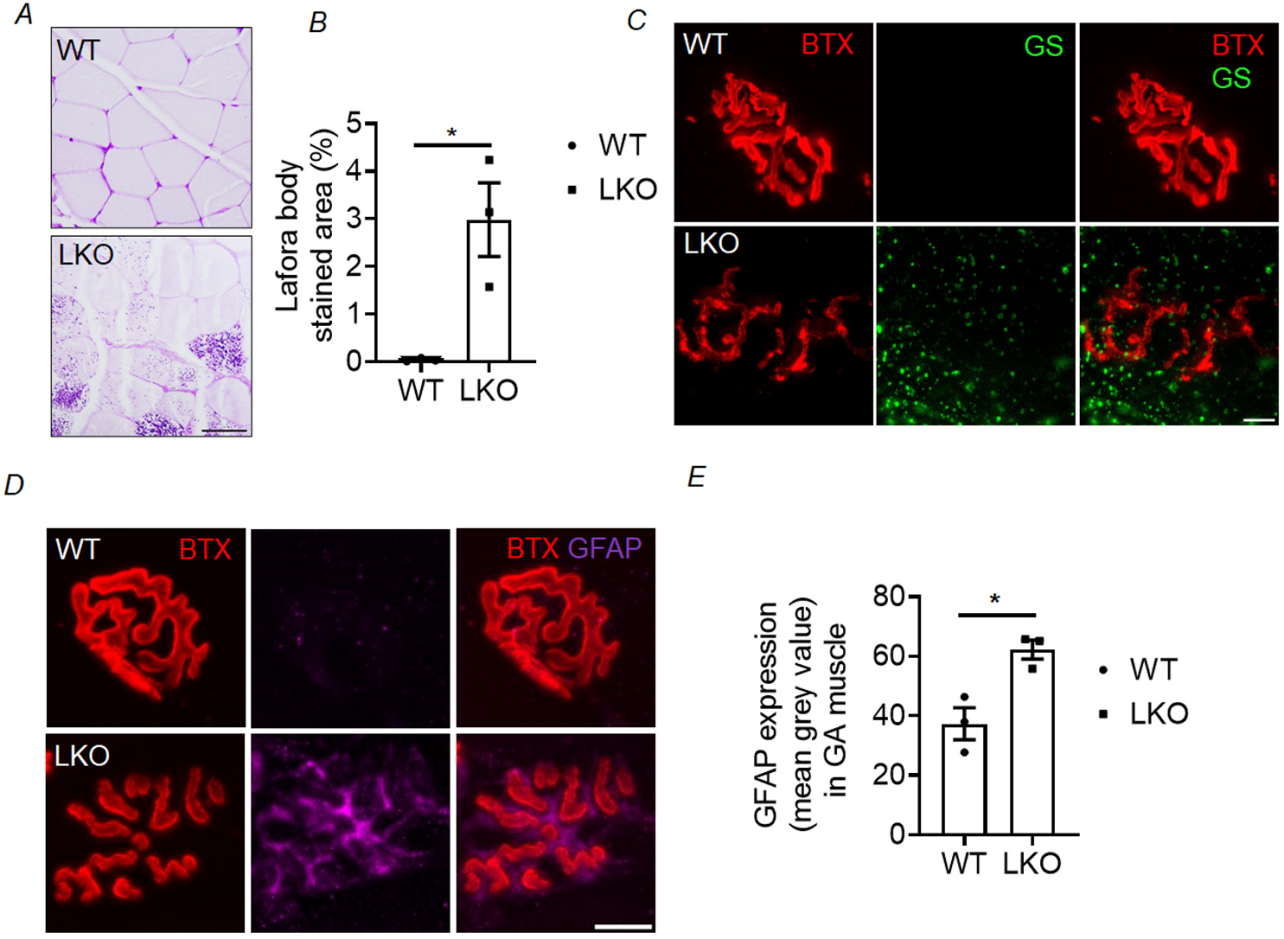
**Accumulation of LBs and increased GFAP expression in the gastrocnemius muscle of the LD mouse model.** (A) Representative images showing PAS-positive LB accumulation in cross-sections of the gastrocnemius muscle of the LKO animals. (B) Percentage of LB-stained area in the gastrocnemius muscle of 10-month-old LKO animals and age-matched WT animals. Each bar represents the mean±s.e.m. [*n*=3 animals for each genotype (WT and LKO); unpaired two-tailed Student's *t-*test; *P<0.05]. (C) Representative images showing GS–BTX co-immunostaining in gastrocnemius muscle of the 10-month-old WT and LKO animals. Note the GS-positive LBs near the endplate region of LKO animals. (D) Representative images from gastrocnemius muscle sections showing GFAP–BTX co-immunostaining in 10-month-old WT and LKO animals. Note the increased GFAP expression (magenta) in the vicinity of the postsynaptic endplate region (BTX, red) in LKO animals. (E) Mean grey value of GFAP^+^ area within the motor endplate in the gastrocnemius (GA) muscle of 10-month-old LKO mice and age-matched WT control mice. Each bar represents the mean±s.e.m.; 50-80 NMJs were analysed per animal, and three animals were used for each genotype (WT and LKO) (unpaired two-tailed Student's *t-*test; *P<0.05). Scale bars: 50 µm (A) and 10 µm (C,D).

In addition to the alterations in the presynaptic and postsynaptic NMJ that may potentially contribute to muscle dysfunction, it is important to consider the possibility of direct involvement of the skeletal muscle in the myopathy phenotype in LD. The accumulation of LBs is recognised as a significant characteristic feature of the LKO mouse model (Ganesh et al., 2002), and LB accumulation was observed in the gastrocnemius muscle in both 10-month-old (Fig. 7A,B) and 5-month-old (Fig. S10A,B) LKO animals. This was further supported by the immunostaining observation of GS-positive LBs near the postsynaptic endplate regions in 10-month-old LKO animals (Fig. 7C). A previous study has indicated a correlation between increased glial cell activity and NMJ dysfunction in a mouse model of amyotrophic lateral sclerosis (ALS) (Liu et al., 2013). Consistent with this notion, we found a significant increase in GFAP expression, specifically within the endplate area of LKO mice of both ages (Fig. 7D,E; Fig. S10C,D).

**Fig. 8.**
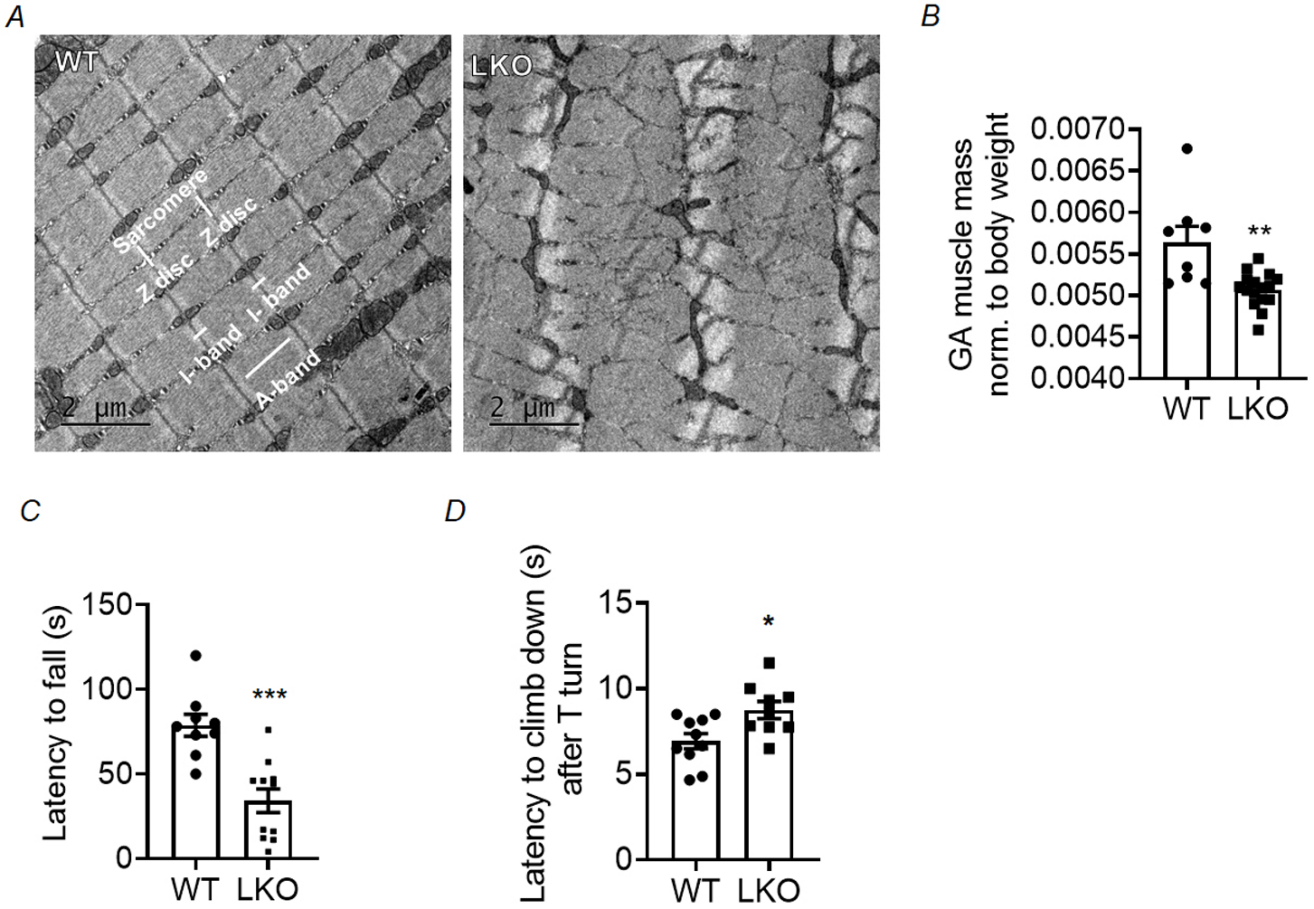
**Ultrastructural alterations in skeletal muscle, loss of muscle mass, and impaired muscle strength and motor performance in the LD mouse model.** (A) Representative TEM images of longitudinal gastrocnemius muscle section illustrating myofibril organisation. Note a well-organized sarcomeric structure in the gastrocnemius muscle of 10-month-old WT animals, with the parallel alignment of the multiple myofibrils with distinguishing features being labeled. The A-band (dark band) consists mainly of thick myosin filaments, whereas the I-band (light band) is composed of thin actin filaments. Each sarcomere is flanked by a Z-disc region. Conversely, a disorganised sarcomeric structure is observed in the gastrocnemius muscle of 10-month-old LKO animals. (B) GA muscle mass normalised to body weight in 10-month-old LKO animals. Each bar represents the mean±s.e.m. (*n*=8 WT and *n*=16 LKO; unpaired two-tailed Student's *t-*tests; **P<0.01). (C,D) Animals were subjected to inverted screen and pole tests to evaluate muscle strength and motor coordination. (C) In the inverted screen test, there was a significant reduction in latency to fall in 10-month-old LKO animals compared to age-matched WT control animals. (D) Similarly, 10-month-old LKO animals took more time than WT animals to climb down to their home cage from the top of the pole, measured as latency to climb down after T turn. Each bar represents the mean±s.e.m. [*n*=9-11 animals for each genotype (WT and LKO); unpaired two-tailed Student's *t-*tests; *P<0.05, ***P<0.001]. Scale bars: 2 µm.

We also examined the ultrastructure of the gastrocnemius muscle in the LKO mouse model. Examination of a longitudinal section of the gastrocnemius muscle using TEM showed a highly organised and repetitive arrangement of myofibrils, characterised by a dense z-line, in WT animals. In contrast, the 10-month-old LKO mice exhibited disorganised myofibrillar patterns and slight z-line streaming, as shown in Fig. 8A. The ultrastructural myopathic changes observed in the 10-month-old LKO mice were accompanied by a notable decrease in the mass of the gastrocnemius muscle compared with that in the age-matched WT controls (Fig. 8B). Nevertheless, there were no significant differences detected in the mass of the gastrocnemius muscle among the 5-month-old LKO animals (Fig. S12A).

Additionally, we evaluated the physiological response of the muscle in the absence of any nerve stimulation to clarify the aetiology of muscle dysfunction in LKO mice, specifically whether it is myopathic or neuropathic in nature. For this, electromyographic (EMG) recordings were conducted in the gastrocnemius muscle of LKO mice (Fig. S11A). The WT control mice exhibited triphasic potentials with a mean amplitude of 2113±181.8 µV. In contrast, the knockout (LKO) mice displayed a significantly reduced amplitude of 997.0±149.4 µV. The quantification of EMG data demonstrated a notable decrease in the average amplitude and an augmented proportion of phases and turns in the muscle potential waveforms in the LKO mice compared to age-matched WT controls (Fig. S11B-D).

### Impaired muscle strength and motor coordination in laforin-deficient mice

After noticing myopathic alterations in LKO animals, our next objective was to examine the impact of these alterations on muscle strength and motor coordination. To assess muscle strength, we conducted an inverted screen test to measure the duration it took for the animals to lose their balance and fall (referred to as latency to fall) from the inverted screen. A significant decrease in latency time was observed for the LKO mice (10-month-old) compared to age-matched WT control mice (Fig. 8C). To evaluate motor coordination, we performed the pole test, measuring the time it took for the mice to move from the top of the pole to their home cage. The results of our study showed that 10-month-old LD mice took a significantly longer time to reach their home cage than did the age-matched WT control mice (Fig. 8D). The 5-month-old LKO mice did not show any noticeable functional muscle deficits (Fig. S12B,C).

## DISCUSSION

Considerable research has been dedicated to investigating the fundamental mechanisms and observable symptoms of LD in the CNS. However, the significance of the peripheral nervous system (PNS) in the development of LD pathophysiology has yet to be elucidated. The knowledge gap is substantial, despite conclusive evidence indicating the accumulation of LBs in the PNS, including skeletal muscle tissue (de Graaf et al., 1989; Criado et al., 2012; Parihar et al., 2018). This study, using an established LD mouse model, provides evidence that dysfunction in the NMJ is responsible for the muscle deficiencies observed in LD.

Our study utilised a comprehensive approach to better understand the possible connection between NMJ dysfunction and LD phenotype in the laforin- and malin-deficient mice. We evaluated the integrity of NMJ transmission using electrophysiological methods, specifically by measuring CMAP and RNS. The examination revealed conclusive evidence of abnormalities in the transmission of signals at the NMJ, which was confirmed by a decrease in muscle response to repeated nerve stimulation, particularly at higher stimulation frequencies. The impairment in NMJ transmission was apparent in the LD mouse models at 5 months of age. Previous studies in aged rodents (Chugh et al., 2020; Fogarty et al., 2019) have shown a similar frequency-dependent worsening of NMJ transmission. The morphological data we obtained on the NMJ of the laforin-deficient mice confirmed and supported our electrophysiological findings regarding changes in both the presynaptic and postsynaptic regions. Consistent with previous findings on age-associated changes in the NMJ of rodents (Courtney and Steinbach, 1981; Rosenheimer, 1990b), we have discovered significant structural abnormalities at the NMJ in the gastrocnemius muscle of LKO mice. Specifically, we observed a decrease in the size and density of the postsynaptic endplate and an increase in fragmentation. Additionally, there was an increase in the number of dispersed junctions, which suggests a reduction in the complexity of the NMJ.

The interaction between the nerve terminal and the motor endplate is a crucial determinant of the functionality of the NMJ. Mounting evidence indicates that denervation or the loss of nerve terminals can result in the fragmentation of the NMJ (Labovitz et al., 1984; Xu and Salpeter, 1997). Our study showed a substantial decrease in fully innervated junctions in the LKO mice. These NMJ morphological findings correlate with a previous study investigating the neuropathic cause of Pompe disease, another type of glycogen storage disorder (Falk et al., 2015). The efficient transmission of signals between neurons and muscles relies on an intricate interaction of various molecular factors that control both the structure and function of the NMJ. Intriguingly, the AGRN/LRP4/MUSK pathway has been extensively investigated as a critical regulatory mechanism that controls the clustering of AchRs at the motor endplates (Glass and Yancopoulos, 1997; Kim et al., 2008; Bolliger et al., 2010; Zong et al., 2012). Disruption in the signalling pathways associated with the development and function of the NMJ results in a range of neuromuscular disorders characterised by muscle weakness and fatigue, such as congenital myasthenic syndromes (Finsterer, 2019). Our study suggests that the decrease in the expression of some of the genes associated with nerve–muscle communication at the NMJ may also contribute to the changes in NMJ structure and function in LD. Clearly, additional investigation is necessary to determine whether there is a direct functional link between laforin/malin proteins and the AGRN/LRP4/MUSK pathway to understand the cause-and-effect relationship between NMJ dysfunction and LD.

It was intriguing to observe in the LD mice that, despite evidence of loss of nerve innervation, there was no change in the CMAP output of the gastrocnemius muscle after a single nerve stimulation. However, a decrease in CMAP response was observed after RNS, especially at higher frequencies. One reason for this is the occurrence of collateral sprouting at the NMJ. Collateral sprouting is a regenerative response of axons, where new connections grow from intact neurons to reinnervate nearby denervated endplates. Collateral sprouting, a compensatory phenomenon, has been observed in various neurodegenerative disorders involving NMJ dysfunction. These disorders include ALS (Fischer et al., 2004; Bruneteau et al., 2015; Jensen et al., 2016), spinal muscular atrophy (Arnold et al., 2021) and Charcot-Marie-Tooth disease. In these conditions, collateral sprouting occurs alongside a reduction in motor units but with increases in motor unit size and fibre type grouping (Jensen et al., 2016). Therefore, it is logical to hypothesise that even though LKO animals experience a loss of motor neuron connectivity, the process of collateral sprouting would have caused an increase in the size of motor units, ultimately resulting in the preservation of CMAP output. This phenomenon is further demonstrated by the observation that the amplitude of CMAP remains within normal ranges in extensively chronic conditions, such as polio, even in the presence of a limited number of remaining motor units but a significantly high innervation ratio (Barkhaus et al., 2024). Furthermore, the current study demonstrates that collateral sprouting has the potential to facilitate the restoration of CMAP levels to normal levels in LD mice. However, newly sprouted axon terminals may exhibit instability in neuromuscular transmission. Rochel and Robbins (1988), using *in vitro* electrophysiology experiments, demonstrated a decrease in neurotransmitter release in newly formed axonal terminals. This reduction in quantal content ultimately decreases the safety factor and consequently leads to unstable neuromuscular transmission in sprouted motor units. Therefore, the loss of NMJ transmission fidelity, as indicated by the CMAP decremental findings upon RNS, may be caused by the diminished safety factor of the sprouted motor units.

Furthermore, it is expected that there will be a decline in NMJ transmission with the advancement of age (Kwon and Yoon, 2017). Nevertheless, we did not detect any notable age-dependent decline in NMJ function in LD animals. These findings align with previous reports on ALS and spinal muscular atrophy patients, which also found no correlation between the extent of CMAP decrement and the duration of the disease (Arnold et al., 2021). However, LKO animals in the older age group did show a decrease in muscle strength, which could be due to a lack of nerve regeneration response that may result in the inability of the muscle to maintain its function in later stages. This was additionally supported by the gradual decrease in innervation observed with increased age in LD animals. Concurrently, with the reduction in functional NMJs, there was a decrease in muscle mass that was dependent on age. Therefore, it is reasonable to suggest that the combined effect of dysfunctional NMJs and muscle atrophy could explain the observed decrease in muscle strength in LD animals.

Recent studies have demonstrated that aging muscle fibres experience recurring processes of denervation and re-innervation (Narici and Maffulli, 2010; Jang and Van Remmen, 2011). This leads to the reorganisation of motor units, which entails the selective elimination of nerve connections in fast-twitch type II muscle fibres, followed by the establishment of new connections through the growth of axons from slow motor neurons. This process leads to the transformation of type II fast fibres into type I slow fibres (Jang and Van Remmen, 2011). Previous research has shown that the accumulation of LBs in skeletal muscle is specific to certain types of muscle fibres, particularly type II (B) fibres, and this accumulation may contribute to the selective loss of these fibres in LD (Yokoi et al., 1968; Turnbull et al., 2016). However, it has yet to be determined whether the axonal sprouting observed in the current study exhibits specificity towards a particular fibre type or not. Nonetheless, collectively, these results indicate that compensatory collateral sprouting may serve as an underlying regenerative mechanism that attempts to preserve muscle function up to a certain threshold during the progression of the disease in the LD mouse model. As the disease progresses, there is a possibility that the reconnection of nerve fibres to previously disconnected junctions may not occur, leading to noticeable muscle dysfunction. Therefore, the regrowth of motor axons and the restoration of nerve–muscle connections could offer a promising treatment option for enhancing motor function recovery (Gordon et al., 2004). Further electrophysiological studies are required to directly evaluate the size of single motor unit potentials and to estimate the number of motor units. Such studies will provide an insight into the motor unit remodelling and offer possible mechanisms involved in functional recovery in LD.

The observed changes in muscle physiology in the LKO mice are additionally supported by the clinical case reports of patients with LD. EMG investigations conducted on individuals diagnosed with LD have revealed notable modifications in spontaneous muscle activity (Driver-Dunkley et al., 2005; Zutt et al., 2016; d'Orsi et al., 2022). In a similar vein, the spontaneous EMG analysis of the gastrocnemius muscle in the LKO mice also demonstrated notable modifications in the muscle potential waveforms, characterised by diminished amplitude and heightened occurrence of polyphasic potentials. Minimal amplitude and polyphasic potentials are predominantly observed in myopathic disorders, although they can also be detected during the initial reinnervation following axonal loss and in disorders involving neuromuscular transmission (Aminoff, 2012).

The present study reveals that the vulnerability of the NMJ to LB accumulation is accompanied by notable alterations at both presynaptic and postsynaptic locations. The number of alpha-motor neurons in the ventral horn region of the spinal cord, which sends primary afferent synaptic inputs to the hindlimb muscles through the sciatic nerve (Jacob, 1998), was significantly decreased in the LKO mouse model. The reduction in alpha-motor neurons can be partially attributed to the abnormal buildup of glycogen in the ventral horn of the lumbar region of the spinal cord. Earlier studies (Brooks et al., 1983; DeRuisseau et al., 2009) have demonstrated the harmful consequences of glycogen accumulation in the spinal cord, resulting in respiratory insufficiency in a mouse model of Pompe disease. Furthermore, a recent study (Brewer et al., 2023) has shown that glycogen tends to accumulate in the spinal cord and brainstem during the symptomatic and advanced stages of ALS. This accumulation of glycogen is strongly associated with reactive astrocytes. Intriguingly, in our study, increased microgliosis and astrogliosis observed in the close vicinity of the ChAT-positive motor neurons could also be driving factors for the loss of motor neurons in the spinal cord in addition to the abnormal glycogen accumulation. This report provides the first evidence linking abnormal glycogen accumulation, increased gliosis and degeneration of alpha-motor neurons in LKO mice. Interestingly, NMJ pathology in LKO animals was accompanied by subtle changes in the sciatic nerve. The decreased G ratio that we observed indicates an increase in myelination of the peripheral nerve, which suggests abnormal morphology of individual axons. This discovery is consistent with the findings of a study conducted by Falk et al. (2015), which also demonstrated an increase in the myelination of axons in the sciatic and phrenic nerves in a mouse model of Pompe disease. Nevertheless, further investigations on nerve conduction velocity are necessary to comprehend the physiological impacts of axon hypermyelination in glycogen storage disorders.

Aside from the neurogenic abnormalities observed at both the presynaptic and postsynaptic sites, it is important to consider the impact of myopathic changes on muscle dysfunction. The presence of elevated GFAP expression near the postsynaptic endplate and the buildup of PAS-positive polyglucosan inclusion bodies in the gastrocnemius muscle suggest their role in the pathophysiology of LD. A prior study (Keller et al., 2009) has demonstrated a notable increase in GFAP expression within Schwann cells of the PNS, which aligns with the clinical manifestations observed in ALS. Further investigation is needed to understand the functional contribution of Schwann cells to the dysfunction of the NMJ in LD.

The UPS is essential for controlling the organisation of muscle sarcomeres (Cohen et al., 2012). TRIM32, an E3-ubiquitin ligase, is known to ubiquitinate actin, desmin, dysbindin and other proteins. Many of these substrates play a role in the regulation of skeletal muscle sarcomeric organisation (Cohen et al., 2012). The TRIM32 knockout mouse, an animal model for limb-girdle muscular dystrophy type 2H, has shown a combination of myopathic and neuropathic changes (Kudryashova et al., 2009). It is worth noting that malin, an E3-ubiquitin ligase mutated in LD, has a similar protein domain structure to that of TRIM32, and that laforin and malin form a functional complex to ubiquitinate its substrates (Chan et al., 2003; Gentry et al., 2005; Garyali et al., 2009). Intriguingly, TRIM32, laforin and malin are known to regulate the UPS, autophagy, glucose metabolism and the WNT signalling pathway (Liu et al., 2006; Garyali et al., 2009; Romá-Mateo et al., 2011; Kumarasinghe et al., 2021). Therefore, it is possible to hypothesise that the laforin–malin complex, either directly or indirectly, regulates the functional properties of the muscle and NMJ-associated proteins. Indeed, NMJ transmission defects have also been observed in the malin-deficient mice in the current study. Clearly, further work is necessary to dissect the molecular pathways leading to NMJ dysfunction in LD.

In summary, our research indicates that dysfunction of the NMJ can occur due to both neuropathic and myopathic changes, ultimately resulting in the loss of muscle function in LD animals. Our study demonstrates the essential contribution of motor neurons, peripheral nerves, skeletal muscle and the NMJ to the development of some of the symptoms of LD. These findings have wider implications for our understanding of the interaction between the CNS and PNS in neurodegenerative disorders and their treatment.

## MATERIALS AND METHODS

### Animals

The research procedure involving animals received approval from the Institutional Animal Ethics Committee (IAEC). The LKO (*Epm2a^−/−^*) mice (Ganesh et al., 2002) and MKO (*Nhlrc1^−/−^*) mice (Turnbull et al., 2010) utilised in the study were obtained from Dr Berge Minassian (Hospital for Sick Children, Toronto, Canada) and bred at our institutional facility. The animals were reared in a controlled environment with a 12-h light and 12-h dark cycle at a constant temperature (22±1°C) and were given *ad libitum* access to food and water. The experimental work utilised homozygous null mutants and age-matched WT littermates, which were 5 and 10 months of age. All experiments ensured sex balance in the experimental groups.

### *In vivo* electrophysiology for assessment of motor unit function

CMAP and RNS measurements were performed on the right hindlimb gastrocnemius muscle following supramaximal sciatic nerve stimulation using a clinical electrodiagnostic device (Nicolet VikingQuest, Natus Neurology Incorporated) as previously described (Arnold et al., 2015; Iyer et al., 2021). In summary, mice were anesthetised using inhaled isoflurane (induction at 5% and maintenance at 2-2.5%; Neon Laboratories Limited). Throughout the procedure, the animal's body temperature was sustained at 37°C by means of a heating pad. A trimmer was used to shave the right hindlimb and posterolateral thigh regions. The mice were positioned in a prone stance, with their hindlimbs fully extended and immobilised using tape. Two insulated monopolar needle electrodes (Natus Neurology Incorporated) were inserted beneath the skin in the region of the sciatic nerve near the proximal hindlimb for the purpose of stimulation. For recording, two high-quality wire ring electrodes (Natus Neurology Incorporated) were utilised. The active electrode was positioned directly on the gastrocnemius muscle at the knee joint, while the reference electrode was placed on the metatarsal region of the foot. A single ground electrode (Natus Neurology Incorporated) was positioned on the tail. To reduce the impedance between the skin and the electrodes, electrode gel was applied to the surface of the electrodes. During CMAP measurements, the sciatic nerve was stimulated using progressively higher current intensities until the maximum CMAP response was detected. Stimulation was done with a constant current intensity below 10 mA and a pulse duration of 0.1 ms. Recordings were made of the amplitudes of the CMAP from baseline to peak and from peak to peak. In order to investigate the defect in NMJ transmission, RNS was conducted (Mori et al., 2012). This involved delivering a series of ten stimuli at a strength greater than that required to elicit a maximal response at frequencies ranging from 10 Hz to 50 Hz. RNS decrement was calculated by measuring the percentage of decrease in CMAP amplitude between the first and the tenth response (Mori et al., 2012).

### EMG

Spontaneous EMG recordings were performed on WT controls and LKO mice at 10 months of age. Data were acquired using the Intan RHS stim/recording system (Intan Technologies). The signals were digitised at 30 kHz. A notch filter with a cutoff frequency of 50 Hz was used to filter out any electrical noise signals at this frequency for online real-time visualisation. A concentric needle electrode was inserted in the right gastrocnemius muscle in an anesthetised mouse to record spontaneous muscle EMG activity. Quantification of EMG activity was performed in terms of amplitude, duration, rise time, number of phases, and turns in the muscle potential waveforms.

### Immunofluorescence staining

Mice were transcardially perfused with 0.9% saline solution. The left gastrocnemius muscles were fixed for 15 min using a 2% paraformaldehyde (PFA) solution for NMJ immunostaining. Afterward, they were rinsed three times with 1× PBS for 10 min each. Cryoblocks were created by subjecting the tissues to cryoprotection in a 30% sucrose solution overnight at a temperature of 4°C using a Poly Freeze tissue freezing medium (SHH0026, Sigma-Aldrich, India). Sections of the mid-belly were obtained by cutting 30 μm slices using a cryostat (Leica Biosystems, India). These slices were then stored at −80 ◦C until preparation for histological staining. For NMJ immunostaining, the sections were initially defrosted at room temperature for 15 min and then rinsed twice for 10 min each in 1× PBS. The sections were treated with 1× PBT (1× PBS with 0.2% Triton X-100) for 10 min to make them permeable. Then, they were incubated in a blocking buffer (5% donkey serum, 0.2% bovine serum albumin in 1× PBT) for 2 h. Subsequently, the sections were exposed to primary antibodies against mouse anti-neurofilament (2H3, Developmental Studies Hybridoma Bank) at a dilution of 1:50 and rabbit anti-synapsin-1 (5297, Cell Signaling Technology) at a dilution of 1:200. This incubation step was carried out overnight at 4°C in the blocking buffer to label the presynaptic side. Following a 3×10 min wash in 1× PBS, the sections were exposed to a secondary antibody solution. This solution consisted of FITC-conjugated donkey anti-rabbit (711-095-152, Jackson ImmunoResearch) and FITC-conjugated donkey anti-mouse (715-095-150, Jackson ImmunoResearch) at a dilution of 1:400, as well as Alexa Fluor 594-conjugated BTX (B13423, Invitrogen, Thermo Fisher Scientific) at a dilution of 1:1000. Incubation took place for 1 h at room temperature. The slides were subsequently rinsed in 1× PBS for a total of three washes, with 10 min intervals for each wash, and then stained with 4′,6-diamidino-2-phenylindole (DAPI; D9542, Sigma Aldrich, India) to specifically mark the cell nuclei. The slides were subsequently prepared with DABCO (Sigma-Aldrich, India) and examined using an AxioCam MRm camera (Zeiss, India) attached to an ObserverZ.1 microscope (Carl Zeiss, India). Zenblue software (Zeiss, India) was used to acquire *z*-stack images with a *z*-step size of 1 µm.

The spinal cord and muscle tissues were immersed in a 4% PFA solution for 24 h to facilitate the staining of motor neurons, GS, astrocytes and microglial cells. Following fixation, the tissues were submerged in a 30% sucrose solution at a temperature of 4°C for the duration of the night. The tissues were cryopreserved using PolyFreeze Tissue Freezing Medium (SHH0026, Sigma-Aldrich, India). The lumbar region of the spinal cord was dissected, and 30 μm coronal sections were taken from the distal end using a cryostat. The sections underwent three washes in 1× Tris-buffered saline (TBS) for 5 min each, followed by blocking with a solution containing 10% donkey serum and 0.2% Triton X-100 at room temperature. The sections were exposed to primary antibodies, including goat anti-ChAT (AB144P, Sigma-Aldrich, India) at a dilution of 1:100, rabbit anti-GFAP antibody (ab7260, Abcam) at a dilution of 1:500, rabbit anti-Iba1 antibody (ab178846, Abcam) at a dilution of 1:500 and rabbit anti-GS (ab40810, Abcam) at a dilution of 1:200. Incubation was carried out for 16-72 h at a temperature of 4°C. After being washed in 1× TBS for three 10 min intervals, the sections were incubated with the following secondary antibodies: Cy3 donkey anti-goat IgG (705-165-003, Jackson ImmunResearch; 1:1000), biotinylated donkey anti-rabbit (711-065-152, Jackson ImmunoResearch; 1:400), Cy3-streptavidin (016-160-084, Jackson ImmunoResearch; 1:400) and Cy5-donkey anti-rabbit (711-175-152, Jackson ImmunoResearch; 1:1000). This incubation took place in a blocking buffer for 1 h at room temperature. The sections were subsequently rinsed in 1× TBS for a total of three 10 min intervals and then treated with DAPI (D9542, Sigma-Aldrich, India) to mark the cell nuclei. The muscle samples underwent GFAP staining using a comparable protocol, with the exception that frozen sections were placed on slides. The sections were subsequently treated with DABCO and examined using a fluorescence microscope (Axio observer 2.0, Carl Zeiss) with Apotome module.

### NMJ imaging and quantification

The NMJs were visualised using a 40× oil immersion objective on an Axiovision epifluorescence microscope equipped with the ApoTome module (Zeiss, India). A total of 70-100 NMJs were randomly observed from three to four sections per animal. Quantification of the *z*-stack was performed using ImageJ software, employing the maximum intensity projection method, as outlined in Falk et al. (2015) and Balch et al. (2021). The area of the postsynaptic endplate was quantified by delineating the boundary of the BTX-stained region using the ‘Freehand selection tool’. The innervation status of the NMJ (fully innervated, partially innervated, denervated) was manually quantified as described (Spaulding et al., 2016; Iyer et al., 2021). Each NMJ synapse was classified as fully innervated if there was complete colocalisation between the presynaptic terminal (marked by synapsin) and the postsynaptic AchR junction (marked by BTX). If the colocalisation was not complete, it was considered partially innervated. If there was no nerve terminal associated with the postsynaptic endplate, it was classified as denervated. The qualitative analysis of NMJ fragmentation was conducted by examining the morphology of the postsynaptic endplate, which was labelled using BTX staining. A solid pretzel-shaped endplate was classified as a non-fragmented NMJ, while an endplate with four or more distinct clusters was categorised as a fragmented NMJ.

### EM

The resin block preparation protocol was followed according to the instructions provided by Lee and Li (2014). In brief, mice were perfused through the heart with a solution of 0.1 M sodium cacodylate buffer, pH 7.4 (C0250, Sigma-Aldrich, India), followed by a solution of EM fixative (2% PFA, 3% glutaraldehyde in 0.1 M cacodylate buffer). The gastrocnemius muscles and sciatic nerves were dissected and then subjected to overnight fixation using the same EM fixative at room temperature. The muscles were treated with a solution containing 1% osmium tetraoxide (201030, Sigma-Aldrich, India) and 1% potassium ferrocyanide in a 0.1 M sodium cacodylate buffer with a pH of 7.4 for a duration of 5 h. In addition, tissue staining was conducted by immersing the tissue in a solution of 2% uranyl acetate for a duration of 2 h. Subsequently, the tissues underwent dehydration by exposure to increasing concentrations of ethanol (25%, 50%, 75%, 95%) and acetone (100%). Each of these steps was performed for a duration of 15 min and repeated twice. Finally, the tissue samples were subjected to resin infiltration using increasing concentrations of resin (45359, Sigma-Aldrich, India). This involved treating the samples with 1/3 resin in acetone overnight, followed by 2/3 resin in acetone for 10 h and, lastly, immersing them in full resin overnight. Finally, the tissues were placed into a silicon mould filled with resin and subjected to polymerisation at a temperature of 60°C for a duration of 72 h. The acquisition of EM images was performed using 70 nm sections obtained from Powertome PC on copper grids (Ted Pella) with the assistance of an FEI Technai G2 12 Twin TEM 120 kV.

### RNA extraction and qRT-PCR

Total RNA was isolated from the gastrocnemius muscles by homogenising them in Trizol reagent (15596026, Ambion, Life Technologies), dissolved in autoclaved RNAse-free water, and processed for cDNA using reverse transcriptase (Thermo Fisher Scientific, India). A reverse transcription reaction was carried out as described in Sinha et al. (2021a,b). The primer sequences used for the PCR amplification are listed in Table S5 (Pratt et al., 2013).

### PAS staining

PAS staining on tissues was performed as previously described (Mitsuno et al., 1999; Ganesh et al., 2002). PFA-fixed tissues were embedded in paraffin (in the case of muscle) or PolyFreeze Tissue Freezing Medium (for the spinal cord). Briefly, paraffin-embedded sections of 7 µm thickness were deparaffinised in xylene and hydrated in a graded series of ethanol (100%, 95%, 75%, 50%). For cryosections of the spinal cord, 1× PBS was used for washing (3×5 min). Next, sections were treated with 1 mg/ml alpha-amylase (28588, Sisco Research Laboratories, India) at 37°C for 5 min. The sections were oxidised with a periodic acid solution (1% w/v) for 20 min at room temperature and rinsed three times with distilled water. The sections were then incubated with Schiff's reagent (29120, S.D. Fine Chem Limited) for 10 min at room temperature and then washed with water. They were subsequently dehydrated and mounted using DPX (06522, Sigma-Aldrich, India), and imaged using a Nikon brightfield microscope at 40×.

### Toluidine Blue staining of resin-embedded sections

The sciatic nerve was harvested following transcardial perfusion of animals. Toluidine Blue staining was performed as described by Ghnenis et al. (2018). In brief, the sciatic nerve was fixed in place by applying Trump's fixative solution (4% formaldehyde, 1% glutaraldehyde in 1× PBS with 1.16 g NaH_2_PO_4_·H_2_O per 100 ml) for a duration of 10 min. After fixation, the nerves were exposed to a 2% solution of freshly prepared osmium tetraoxide (201030, Sigma-Aldrich, India) diluted in Trump's fixative for 2 h at room temperature. Nerve segments were washed twice using 1× PBS for 10 min to remove osmium tetraoxide and Trump's solution. Nerve segments were then dehydrated using increasing concentrations of acetone (30%, 60%, 90%) for 10 min each and then in 100% acetone three times for 10 mins each. After the dehydration process, the nerve segments were submerged in a solution consisting of equal parts of the final epoxy embedding mixture (45359, Sigma-Aldrich, India) and 100% acetone for 30 min. This was followed by immersing the nerve segments in a solution consisting of two parts of the final embedding mixture and one part 100% acetone for another 30 min. Finally, the nerve segments were soaked in the final embedding mixture for an additional 30 min. The segments were subsequently placed in a silicone mould containing the epoxy mixture and left in a 60°C oven overnight. After polymerisation, an ultramicrotome was used to cut the semi-thin (500 nm) nerve cross-sections. The sections were subsequently stained with a 1% solution of Toluidine Blue. Excess Toluidine Blue solution was rinsed off by dipping the slides into deionised water three to four times. The slides were air dried overnight at ambient temperature and subsequently coverslipped with DPX. The mounted sections were observed using a Nikon Eclipse 80i bright-light microscope at a magnification of 100×. Myltrace software (Kaiser et al., 2021) was used to calculate the G ratio for quantifying myelin thickness.

### Inverted screen test

An inverted screen test was performed to assess the muscle strength in LKO and age-matched WT mice. It was performed as described previously (Kondziella, 1964; Sango et al., 1996; Ganesh et al., 2002). Mice were placed in the middle of the modified mesh cover of the cage, which was immediately turned 180°. The time to fall off the mesh cover was recorded as latency to fall (s). Each mouse underwent three trials with a maximum trial time of 120 s.

### Pole test

The pole test was performed to assess the motor coordination in mice as previously described (Freitag et al., 2003). Briefly, the animals were positioned on the top of the rod so that they grasped the rod with four paws and their heads pointing upwards. The latency to climb down to their home cage was then recorded. In addition, the ability of the mouse to turn 180° and walk down with its head pointed downwards was recorded as time to T turn (s). Each mouse underwent three consecutive trials with a resting period of 30 s between every trial.

### Statistical analysis

Statistical analysis was performed using GraphPad Software, Prism 8 (GraphStats Technologies, India). Data are expressed as mean±s.e.m. from at least three independent experiments. For the analyses of RNS (genotype and frequency as fixed factors, and CMAP decrement as a dependent factor), NMJ innervation (genotype and innervation category as fixed factors, and percentage of NMJs with a particular innervation pattern as a dependent factor) and microglial morphology (genotype and morphological phenotype as fixed factors, and percentage of microglial cells with a particular morphology as a dependent factor), two-way ANOVA with Sidak's multiple comparison was used. The remaining data were analysed using unpaired two-tailed Student's *t*-test (when comparing two groups, i.e. WT and LKO) or one-way ANOVA (when comparing more than two groups, i.e. WT, LKO, and MKO). Differences were considered to be significant at *P*<0.05.

## Supplementary Material

10.1242/dmm.050905_sup1 Supplementary information

## References

[DMM050905C1] Aguado, C., Sarkar, S., Korolchuk, V. I., Criado, O., Vernia, S., Boya, P., Sanz, P., De Córdoba, S. R., Knecht, E. and Rubinsztein, D. C. (2010). Laforin, the most common protein mutated in Lafora disease, regulates autophagy. *Hum. Mol. Genet.* 19, 2867-2876. 10.1093/hmg/ddq190 20453062 PMC2893813

[DMM050905C99] Al Mufargi, Y., Qureshi, A. and Al Asmi, A. (2020). Lafora disease: report of a rare entity. *Cureus* 12, e6793. 10.7759/cureus.6793 32140352 PMC7046017

[DMM050905C2] Aminoff, M. J. (2012). Clinical electromyography. In: *Electrodiagnosis in Clinical Neurology*, pp. 233-259. Philadelphia: Churchill Livingstone.

[DMM050905C3] Anraku, S. and Kawasaki, H. (1966). A histochemical study on myoclonus-epilepsy (Lafora-body type). *Folia Psychiatr. Neurol. Jpn.* 20, 33-43. 10.1111/j.1440-1819.1966.tb00057.x 6013333

[DMM050905C4] Arnold, W. D., Sheth, K. A., Wier, C. G., Kissel, J. T., Burghes, A. H. and Kolb, S. J. (2015). Electrophysiological motor unit number estimation (MUNE) measuring compound muscle action potential (CMAP) in mouse hindlimb muscles. *J. Vis. Exp.* 103, 52899. 10.3791/52899 PMC4676269 26436455

[DMM050905C5] Arnold, W. D., Severyn, S., Zhao, S., Kline, D., Linsenmayer, M., Kelly, K., Tellez, M., Bartlett, A., Heintzman, S., Reynolds, J. et al. (2021). Persistent neuromuscular junction transmission defects in adults with spinal muscular atrophy treated with nusinersen. *BMJ Neurol. Open* 3, e000164. 10.1136/bmjno-2021-000164 PMC8362737 34466806

[DMM050905C6] Balch, M. H. H., Harris, H., Chugh, D., Gnyawali, S., Rink, C., Nimjee, S. M. and Arnold, W. D. (2021). Ischemic stroke-induced polyaxonal innervation at the neuromuscular junction is attenuated by robot-assisted mechanical therapy. *Exp. Neurol.* 343, 113767. 10.1016/j.expneurol.2021.113767 34044000 PMC8286354

[DMM050905C7] Barkhaus, P. E., Nandedkar, S. D., De Carvalho, M., Swash, M. and Stålberg, E. V. (2024). Revisiting the compound muscle action potential (CMAP). *Clin. Neurophysiol. Pract.* 9, 176-200. 10.1016/j.cnp.2024.04.002 38807704 PMC11131082

[DMM050905C8] Bolliger, M. F., Zurlinden, A., Lüscher, D., Bütikofer, L., Shakhova, O., Francolini, M., Kozlov, S. V., Cinelli, P., Stephan, A., Kistler, A. D. et al. (2010). Specific proteolytic cleavage of agrin regulates maturation of the neuromuscular junction. *J. Cell Sci.* 123, 3944-3955. 10.1242/jcs.072090 20980386

[DMM050905C9] Brewer, M. K. and Gentry, M. S. (2019). Brain glycogen structure and its associated proteins: past, present. In: *Brain Glycogen Metabolism* (ed. M. DiNuzzo and A. Schousboe), pp. 17-81. Switzerland: Springer Nature. 10.1007/978-3-030-27480-1_2 PMC7239500 31667805

[DMM050905C10] Brewer, M. K., Putaux, J. L., Rondon, A., Uittenbogaard, A., Mitchell, A. S. and Gentry, M. S. (2020). Polyglucosan body structure in Lafora disease. *Carbohydr. Polym.* 240, 116260. 10.1016/j.carbpol.2020.116260 32475552 PMC7266828

[DMM050905C11] Brewer, M. K., Torres, P., Ayala, V., Portero-Otin, M., Pamplona, R., Andrés-Benito, P., Ferrer, I., Gentry, M. S., Guinovart, J. J. and Duran, J. (2023). Glycogen accumulation modulates life span in a mouse model of amyotrophic lateral sclerosis. *J. Neurochem.* 168, 744-759. 10.1111/jnc.15906 37401737 PMC10764643

[DMM050905C12] Brooks, B. R., Feussner, G. K. and Lust, W. D. (1983). Spinal cord metabolic changes in murine retrovirus-induced motor neuron disease. *Brain Res. Bull.* 11, 681-686. 10.1016/0361-9230(83)90011-4 6318918

[DMM050905C13] Bruneteau, G., Bauché, S., Gonzalez De Aguilar, J. L., Brochier, G., Mandjee, N., Tanguy, M.-L., Hussain, G., Behin, A., Khiami, F., Sariali, E. et al. (2015). Endplate denervation correlates with Nogo-A muscle expression in amyotrophic lateral sclerosis patients. *Ann. Clin. Transl. Neurol.* 2, 362-372. 10.1002/acn3.179 25909082 PMC4402082

[DMM050905C14] Chan, E. M., Young, E. J., Ianzano, L., Munteanu, I., Zhao, X., Christopoulos, C. C., Avanzini, G., Elia, M., Ackerley, C. A., Jovic, N. J. et al. (2003). Mutations in NHLRC1 cause progressive myoclonus epilepsy. *Nat. Genet.* 35, 125-127. 10.1038/ng1238 12958597

[DMM050905C15] Chugh, D., Iyer, C. C., Wang, X., Bobbili, P., Rich, M. M. and Arnold, W. D. (2020). Neuromuscular junction transmission failure is a late phenotype in aging mice. *Neurobiol. Aging* 86, 182-190. 10.1016/j.neurobiolaging.2019.10.022 31866157 PMC6995686

[DMM050905C100] Cohen, S., Zhai, B., Gygi, S. P. and Goldberg, A. L. (2012). Ubiquitylation by Trim32 causes coupled loss of desmin, Z-bands, and thin filaments in muscle atrophy. *J. Cell Biol.* 198, 575-589. 10.1083/jcb.201110067 22908310 PMC3514026

[DMM050905C16] Coleman, D. L., Gambetti, P., Mauro, S. D. and Blume, R. E. (1974). Muscle in Lafora disease. *Arch. Neurol.* 31, 396-406. 10.1001/archneur.1974.00490420062007 4140718

[DMM050905C17] Courtney, J. and Steinbach, J. H. (1981). Age changes in neuromuscular junction morphology and acetylcholine receptor distribution on rat skeletal muscle fibres. *J. Physiol.* 320, 435-447. 10.1113/jphysiol.1981.sp013960 7320945 PMC1244058

[DMM050905C18] Criado, O., Aguado, C., Gayarre, J., Duran-Trio, L., Garcia-Cabrero, A. M., Vernia, S., San Millán, B., Heredia, M., Romá-Mateo, C., Mouron, S. et al. (2012). Lafora bodies and neurological defects in malin-deficient mice correlate with impaired autophagy. *Hum. Mol. Genet.* 21, 1521-1533. 10.1093/hmg/ddr590 22186026

[DMM050905C19] De Graaf, A. S., Ancker, E., Rutherfoord, G. S., Van Der Walt, J. J. and Rossouw, D. J. (1989). Lafora-body disease with optic atrophy, macular degeneration and cardiac failure. *J. Neurol. Sci.* 93, 69-84. 10.1016/0022-510x(89)90162-7 2509638

[DMM050905C20] Depaoli-Roach, A. A., Tagliabracci, V. S., Segvich, D. M., Meyer, C. M., Irimia, J. M. and Roach, P. J. (2010). Genetic depletion of the malin E3 ubiquitin ligase in mice leads to lafora bodies and the accumulation of insoluble laforin. *J. Biol. Chem.* 285, 25372-25381. 10.1074/jbc.M110.148668 20538597 PMC2919100

[DMM050905C21] Deruisseau, L. R., Fuller, D. D., Qiu, K., Deruisseau, K. C., Donnelly, W. H., Mah, C., Reier, P. J. and Byrne, B. J. (2009). Neural deficits contribute to respiratory insufficiency in Pompe disease. *Proc. Natl. Acad. Sci. USA* 106, 9419-9424. 10.1073/pnas.0902534106 19474295 PMC2695054

[DMM050905C22] d'Orsi, G., Di Claudio, M. T., Palumbo, O. and Carella, M. (2022). Electro-clinical features and management of the late stage of Lafora disease. *Front. Neurol.* 13, 969297. 10.3389/fneur.2022.969297 36277909 PMC9580008

[DMM050905C23] Driver-Dunkley, E., Sirven, J., Drazkowski, J. and Caviness, J. N. (2005). Lafora disease with primary generalized epileptic myoclonus. *Mov. Disord.* 20, 907-911. 10.1002/mds.20523 15929095

[DMM050905C24] Duran, J., Gruart, A., García-Rocha, M., Delgado-García, J. M. and Guinovart, J. J. (2014). Glycogen accumulation underlies neurodegeneration and autophagy impairment in Lafora disease. *Hum. Mol. Genet.* 23, 3147-3156. 10.1093/hmg/ddu024 24452334

[DMM050905C25] Falk, D. J., Todd, A. G., Lee, S., Soustek, M. S., Elmallah, M. K., Fuller, D. D., Notterpek, L. and Byrne, B. J. (2015). Peripheral nerve and neuromuscular junction pathology in Pompe disease. *Hum. Mol. Genet.* 24, 625-636. 10.1093/hmg/ddu476 25217571 PMC4291243

[DMM050905C26] Finsterer, J. (2019). Congenital myasthenic syndromes. *Orphanet J. Rare Dis.* 14, 57. 10.1186/s13023-019-1025-5 30808424 PMC6390566

[DMM050905C27] Fischer, L. R., Culver, D. G., Tennant, P., Davis, A. A., Wang, M., Castellano-Sanchez, A., Khan, J., Polak, M. A. and Glass, J. D. (2004). Amyotrophic lateral sclerosis is a distal axonopathy: evidence in mice and man. *Exp. Neurol.* 185, 232-240. 10.1016/j.expneurol.2003.10.004 14736504

[DMM050905C28] Fogarty, M. J., Gonzalez Porras, M. A., Mantilla, C. B. and Sieck, G. C. (2019). Diaphragm neuromuscular transmission failure in aged rats. *J. Neurophysiol.* 122, 93-104. 10.1152/jn.00061.2019 31042426 PMC6689786

[DMM050905C29] Freitag, S., Schachner, M. and Morellini, F. (2003). Behavioral alterations in mice deficient for the extracellular matrix glycoprotein tenascin-R. *Behav. Brain Res.* 145, 189-207. 10.1016/s0166-4328(03)00109-8 14529817

[DMM050905C30] Ganesh, S., Agarwala, K. L., Ueda, K., Akagi, T., Shoda, K., Usui, T., Hashikawa, T., Osada, H., Delgado-Escueta, A. V. and Yamakawa, K. (2000). Laforin, defective in the progressive myoclonus epilepsy of Lafora type, is a dual-specificity phosphatase associated with polyribosomes. *Hum. Mol. Genet.* 9, 2251-2261. 10.1093/oxfordjournals.hmg.a018916 11001928

[DMM050905C31] Ganesh, S., Delgado-Escueta, A. V., Sakamoto, T., Avila, M. R., Machado-Salas, J., Hoshii, Y., Akagi, T., Gomi, H., Suzuki, T. and Amano, K. (2002). Targeted disruption of the Epm2a gene causes formation of Lafora inclusion bodies, neurodegeneration, ataxia, myoclonus epilepsy and impaired behavioral response in mice. *Hum. Mol. Genet.* 11, 1251-1262. 10.1093/hmg/11.11.1251 12019206

[DMM050905C32] Ganesh, S., Puri, R., Singh, S., Mittal, S. and Dubey, D. (2006). Recent advances in the molecular basis of Lafora's progressive myoclonus epilepsy. *J. Hum. Genet.* 51, 1-8. 10.1007/s10038-005-0321-1 16311711

[DMM050905C33] Garyali, P., Siwach, P., Singh, P. K., Puri, R., Mittal, S., Sengupta, S., Parihar, R. and Ganesh, S. (2009). The malin-laforin complex suppresses the cellular toxicity of misfolded proteins by promoting their degradation through the ubiquitin-proteasome system. *Hum. Mol. Genet.* 18, 688-700. 10.1093/hmg/ddn398 19036738

[DMM050905C34] Garyali, P., Segvich, D. M., Depaoli-Roach, A. A. and Roach, P. J. (2014). Protein degradation and quality control in cells from laforin and malin knockout mice. *J. Biol. Chem.* 289, 20606-20614. 10.1074/jbc.M114.580167 24914213 PMC4110273

[DMM050905C35] Gentry, M. S., Worby, C. A. and Dixon, J. E. (2005). Insights into Lafora disease: malin is an E3 ubiquitin ligase that ubiquitinates and promotes the degradation of laforin. *Proc. Natl. Acad. Sci. USA* 102, 8501-8506. 10.1073/pnas.0503285102 15930137 PMC1150849

[DMM050905C36] Ghnenis, A. B., Czaikowski, R. E., Zhang, Z. J. and Bushman, J. S. (2018). Toluidine blue staining of resin-embedded sections for evaluation of peripheral nerve morphology. *J. Vis. Exp.* 137, 58031. 10.3791/58031 PMC6102041 30035773

[DMM050905C37] Glass, D. J. and Yancopoulos, G. D. (1997). Sequential roles of agrin, MuSK and rapsyn during neuromuscular junction formation. *Curr. Opin. Neurobiol.* 7, 379-384. 10.1016/s0959-4388(97)80066-9 9232805

[DMM050905C38] Gordon, T., Hegedus, J. and Tam, S. L. (2004). Adaptive and maladaptive motor axonal sprouting in aging and motoneuron disease. *Neurol. Res.* 26, 174-185. 10.1179/016164104225013806 15072637

[DMM050905C39] Iyer, C. C., Chugh, D., Bobbili, P. J., Iii, A. J. B., Crum, A. E., Yi, A. F., Kaspar, B. K., Meyer, K. C., Burghes, A. H. M. and Arnold, W. D. (2021). Follistatin-induced muscle hypertrophy in aged mice improves neuromuscular junction innervation and function. *Neurobiol. Aging* 104, 32-41. 10.1016/j.neurobiolaging.2021.03.005 33964607 PMC8225567

[DMM050905C40] Jacob, J. M. (1998). Lumbar motor neuron size and number is affected by age in male F344 rats. *Mech. Ageing Dev.* 106, 205-216. 10.1016/s0047-6374(98)00117-1 9883984

[DMM050905C41] Jang, Y. C. and Van Remmen, H. (2011). Age-associated alterations of the neuromuscular junction. *Exp. Gerontol.* 46, 193-198. 10.1016/j.exger.2010.08.029 20854887 PMC3026920

[DMM050905C42] Jensen, L., Jørgensen, L. H., Bech, R. D., Frandsen, U. and Schrøder, H. D. (2016). Skeletal muscle remodelling as a function of disease progression in amyotrophic lateral sclerosis. *Biomed. Res. Int.* 2016, 5930621. 10.1155/2016/5930621 27195289 PMC4852332

[DMM050905C43] Kaiser, T., Allen, H. M., Kwon, O., Barak, B., Wang, J., He, Z., Jiang, M. and Feng, G. (2021). MyelTracer: a semi-automated software for myelin g-ratio quantification. *eNeuro* 8, ENEURO, 0558-20.2021. 10.1523/ENEURO.0558-20.2021 PMC8298095 34193510

[DMM050905C44] Keller, A. F., Gravel, M. and Kriz, J. (2009). Live imaging of amyotrophic lateral sclerosis pathogenesis: disease onset is characterized by marked induction of GFAP in Schwann cells. *Glia* 57, 1130-1142. 10.1002/glia.20836 19115383

[DMM050905C45] Kim, N., Stiegler, A. L., Cameron, T. O., Hallock, P. T., Gomez, A. M., Huang, J. H., Hubbard, S. R., Dustin, M. L. and Burden, S. J. (2008). Lrp4 is a receptor for Agrin and forms a complex with MuSK. *Cell* 135, 334-342. 10.1016/j.cell.2008.10.002 18848351 PMC2933840

[DMM050905C46] Kondziella, W. (1964). A new method for the measurement of muscle relaxation in white mice. *Arch. Int. Pharmacodyn. Ther.* 152, 277-284. 14265648

[DMM050905C47] Kudryashova, E., Wu, J., Havton, L. A. and Spencer, M. J. (2009). Deficiency of the E3 ubiquitin ligase TRIM32 in mice leads to a myopathy with a neurogenic component. *Hum. Mol. Genet.* 18, 1353. 10.1093/hmg/ddp036 19155210 PMC2722196

[DMM050905C48] Kumarasinghe, L., Xiong, L., Garcia-Gimeno, M. A., Lazzari, E., Sanz, P. and Meroni, G. (2021). TRIM32 and malin in neurological and neuromuscular rare diseases. *Cells* 10, 820. 10.3390/cells10040820 33917450 PMC8067510

[DMM050905C49] Kwon, Y. N. and Yoon, S. S. (2017). Sarcopenia: neurological point of view. *J. Bone Metab.* 24, 83. 10.11005/jbm.2017.24.2.83 28642851 PMC5472802

[DMM050905C50] Labovitz, S. S., Robbins, N. and Fahim, M. A. (1984). Endplate topography of denervated and disused rat neuromuscular junctions: comparison by scanning and light microscopy. *Neuroscience* 11, 963-971. 10.1016/0306-4522(84)90207-0 6738862

[DMM050905C51] Lee, Y. I. and Li, Y. (2014). The use of synaptic basal lamina and its components to identify sites of recent morphological alterations at mammalian neuromuscular junctions. In: *Extracellular Matrix, Neuromethods* (ed. J. B. Leach and E. M. Powell), pp. 12-22, Humana Press, Springer.

[DMM050905C52] Lee, C., Seri, O. and Kang, D. B. (2008). Anesthetic management of a patient with Lafora's disease: a case report. *Korean J. Anesthesiol.* 54, S51-S54. 10.4097/kjae.2008.54.3.S51

[DMM050905C53] Liu, Y., Wang, Y., Wu, C., Liu, Y. and Zheng, P. (2006). Dimerization of Laforin is required for its optimal phosphatase activity, regulation of GSK3beta phosphorylation, and Wnt signaling. *J. Biol. Chem.* 281, 34768-34774. 10.1074/jbc.M607778200 16971387

[DMM050905C54] Liu, J.-X., Brännström, T., Andersen, P. M. and Pedrosa-Domellöf, F. (2013). Distinct changes in synaptic protein composition at neuromuscular junctions of extraocular muscles versus limb muscles of ALS donors. *PLoS One* 8, e57473. 10.1371/journal.pone.0057473 23468993 PMC3582511

[DMM050905C55] Marshall, K. L. and Farah, M. H. (2021). Axonal regeneration and sprouting as a potential therapeutic target for nervous system disorders. *Neural Regen. Res.* 16, 1901-1910. 10.4103/1673-5374.308077 33642358 PMC8343323

[DMM050905C56] Minassian, B. A. (2001). Lafora's disease: towards a clinical, pathologic, and molecular synthesis. *Pediatr. Neurol.* 25, 21-29. 10.1016/s0887-8994(00)00276-9 11483392

[DMM050905C57] Mitra, S., Gumusgoz, E. and Minassian, B. A. (2022). Lafora disease: current biology and therapeutic approaches. *Rev. Neurol. (Paris)* 178, 315-325. 10.1016/j.neurol.2021.06.006 34301405 PMC8770683

[DMM050905C58] Mitsuno, S., Takahashi, M., Gondo, T., Hoshii, Y., Hanai, N., Ishihara, T. and Yamada, M. (1999). Immunohistochemical, conventional and immunoelectron microscopical characteristics of periodic acid-Schiff-positive granules in the mouse brain. *Acta Neuropathol.* 98, 31-38. 10.1007/s004010051048 10412798

[DMM050905C101] Mori, S., Kubo, S., Akiyoshi, T., Yamada, S., Miyazaki, T., Hotta, H., Desaki, J., Kishi, M., Konishi, T., Nishino, Y. et al. (2012). Antibodies against muscle-specific kinase impair both presynaptic and postsynaptic functions in a murine model of myasthenia gravis. *Am. J. Pathol.* 180, 798-810. 10.1016/j.ajpath.2011.10.031 22142810

[DMM050905C102] Namba, M. and Ota, T. (1966). The ultrastructure of Lafora body. *Bull. Yamaguchi Med. School* 13, 233-250. https://petit.lib.yamaguchi-u.ac.jp/journals/bulletin/v/13/i/4/item/614

[DMM050905C59] Narici, M. V. and Maffulli, N. (2010). Sarcopenia: characteristics, mechanisms and functional significance. *Br. Med. Bull.* 95, 139-159. 10.1093/bmb/ldq008 20200012

[DMM050905C60] Neville, H. E., Brooke, M. H. and Austin, J. H. (1974). Studies in myoclonus epilepsy. (Lafora body form). IV. Skeletal muscle abnormalities. *Arch. Neurol.* 30, 466-474. 10.1001/archneur.1974.00490360042009 4133238

[DMM050905C61] Parihar, R. and Ganesh, S. (2024). Lafora progressive myoclonus epilepsy: disease mechanism and therapeutic attempts. *J. Biosci.* 49, 22. 10.1007/s12038-023-00407-6 38287677

[DMM050905C62] Parihar, R., Rai, A. and Ganesh, S. (2018). Lafora disease: from genotype to phenotype. *J. Genet.* 97, 611-624. 10.1007/s12041-018-0949-1 30027899

[DMM050905C63] Pratt, S. J. P., Shah, S. B., Ward, C. W., Inacio, M. P., Stains, J. P. and Lovering, R. M. (2013). Effects of in vivo injury on the neuromuscular junction in healthy and dystrophic muscles. *J. Physiol.* 591, 559-570. 10.1113/jphysiol.2012.241679 23109110 PMC3577526

[DMM050905C64] Puri, R. and Ganesh, S. (2010). Laforin in autophagy: a possible link between carbohydrate and protein in Lafora disease? *Autophagy* 6, 1229-1231. 10.4161/auto.6.8.13307 20818153

[DMM050905C65] Puri, R., Suzuki, T., Yamakawa, K. and Ganesh, S. (2012). Dysfunctions in endosomal-lysosomal and autophagy pathways underlie neuropathology in a mouse model for Lafora disease. *Hum. Mol. Genet.* 21, 175-184. 10.1093/hmg/ddr452 21965301

[DMM050905C66] Rochel, S. and Robbins, N. (1988). Effect of partial denervation and terminal field expansion on neuromuscular transmitter release and nerve terminal structure. *J. Neurosci.* 8, 332-338. 10.1523/JNEUROSCI.08-01-00332.1988 2892899 PMC6569365

[DMM050905C67] Romá-Mateo, C., Moreno, D., Vernia, S., Rubio, T., Bridges, T. M., Gentry, M. S. and Sanz, P. (2011). Lafora disease E3-ubiquitin ligase malin is related to TRIM32 at both the phylogenetic and functional level. *BMC Evol. Biol.* 11, 225. 10.1186/1471-2148-11-225 21798009 PMC3160408

[DMM050905C68] Rosenheimer, J. L. (1990a). Factors affecting denervation-like changes at the neuromuscular junction during aging. *Int. J. Dev. Neurosci.* 8, 643-654. 10.1016/0736-5748(90)90059-b 1963025

[DMM050905C69] Rosenheimer, J. L. (1990b). Ultraterminal sprouting in innervated and partially denervated adult and aged rat muscle. *Neuroscience* 38, 763-770. 10.1016/0306-4522(90)90069-g 2270142

[DMM050905C70] Rudenskaia, G. E., Zakharova, E. I., Karpin, S. L. and Uchaev, D. A. (2010). Myoclonic epilepsy of Lafora: a case report. *Zh. Nevrol. Psikhiatr. Im. S. S. Korsakova* 110, 11-16. 20873469

[DMM050905C71] Sango, K., Mcdonald, M. P., Crawley, J. N., Mack, M. L., Tifft, C. J., Skop, E., Starr, C. M., Hoffmann, A., Sandhoff, K., Suzuki, K. et al. (1996). Mice lacking both subunits of lysosomal beta-hexosaminidase display gangliosidosis and mucopolysaccharidosis. *Nat. Genet.* 14, 348-352. 10.1038/ng1196-348 8896570

[DMM050905C72] Schwarz, G. A. and Yanoff, M. (1965). Lafora's disease. Distict clinic-pathologic form of Unverricht's syndrome. *Arch. Neurol.* 12, 172-188. 10.1001/archneur.1965.00460260062008 14237775

[DMM050905C73] Sharma, J., Mukherjee, D., Rao, S. N. R., Iyengar, S., Shankar, S. K., Satishchandra, P. and Jana, N. R. (2013). Neuronatin-mediated aberrant calcium signaling and endoplasmic reticulum stress underlie neuropathology in Lafora disease. *J. Biol. Chem.* 288, 9482-9490. 10.1074/jbc.M112.416180 23408434 PMC3611017

[DMM050905C74] Singh, S. and Ganesh, S. (2009). Lafora progressive myoclonus epilepsy: a meta-analysis of reported mutations in the first decade following the discovery of the EPM2A and NHLRC1 genes. *Hum. Mutat.* 30, 715-723. 10.1002/humu.20954 19267391

[DMM050905C75] Singh, P. K., Singh, S. and Ganesh, S. (2012). The laforin-malin complex negatively regulates glycogen synthesis by modulating cellular glucose uptake via glucose transporters. *Mol. Cell. Biol.* 32, 652-663. 10.1128/MCB.06353-11 22124153 PMC3266598

[DMM050905C76] Singh, P. K., Singh, S. and Ganesh, S. (2013). Activation of serum/glucocorticoid-induced kinase 1 (SGK1) underlies increased glycogen levels, mTOR activation, and autophagy defects in Lafora disease. *Mol. Biol. Cell* 24, 3776-3786. 10.1091/mbc.E13-05-0261 24131995 PMC3861076

[DMM050905C77] Sinha, P., Verma, B. and Ganesh, S. (2021a). Trehalose ameliorates seizure susceptibility in Lafora disease mouse models by suppressing neuroinflammation and endoplasmic reticulum stress. *Mol. Neurobiol.* 58, 1088-1101. 10.1007/s12035-020-02170-3 33094475

[DMM050905C78] Sinha, P., Verma, B. and Ganesh, S. (2021b). Dexamethasone-induced activation of heat shock response ameliorates seizure susceptibility and neuroinflammation in mouse models of Lafora disease. *Exp. Neurol.* 340, 113656. 10.1016/j.expneurol.2021.113656 33639210

[DMM050905C79] Spaulding, E. L., Sleigh, J. N., Morelli, K. H., Pinter, M. J., Burgess, R. W. and Seburn, K. L. (2016). Synaptic deficits at neuromuscular junctions in two mouse models of charcot-marie-tooth type 2d. *J. Neurosci.* 36, 3254-3267. 10.1523/JNEUROSCI.1762-15.2016 26985035 PMC4792937

[DMM050905C80] Sullivan, M. A., Nitschke, S., Steup, M., Minassian, B. A. and Nitschke, F. (2017). Pathogenesis of Lafora disease: transition of soluble glycogen to insoluble polyglucosan. *Int. J. Mol. Sci.* 18, 1743. 10.3390/ijms18081743 28800070 PMC5578133

[DMM050905C81] Tagliabracci, V. S., Turnbull, J., Wang, W., Girard, J.-M., Zhao, X., Skurat, A. V., Delgado-Escueta, A. V., Minassian, B. A., Depaoli-Roach, A. A. and Roach, P. J. (2007). Laforin is a glycogen phosphatase, deficiency of which leads to elevated phosphorylation of glycogen in vivo. *Proc. Natl. Acad. Sci. USA* 104, 19262-19266. 10.1073/pnas.0707952104 18040046 PMC2148278

[DMM050905C82] Tagliabracci, V. S., Girard, J. M., Segvich, D., Meyer, C., Turnbull, J., Zhao, X., Minassian, B. A., Depaoli-Roach, A. A. and Roach, P. J. (2008). Abnormal metabolism of glycogen phosphate as a cause for Lafora disease. *J. Biol. Chem.* 283, 33816-33825. 10.1074/jbc.M807428200 18852261 PMC2590708

[DMM050905C83] Turnbull, J., Wang, P., Girard, J.-M., Ruggieri, A., Wang, T. J., Draginov, A. G., Kameka, A. P., Pencea, N., Zhao, X., Ackerley, C. A. et al. (2010). Glycogen hyperphosphorylation underlies Lafora body formation. *Ann. Neurol.* 68, 925-933. 10.1002/ana.22156 21077101

[DMM050905C84] Turnbull, J., Depaoli-Roach, A. A., Zhao, X., Cortez, M. A., Pencea, N., Tiberia, E., Piliguian, M., Roach, P. J., Wang, P., Ackerley, C. A. et al. (2011). PTG depletion removes Lafora bodies and rescues the fatal epilepsy of Lafora disease. *PLoS Genet.* 7, e1002037. 10.1371/journal.pgen.1002037 21552327 PMC3084203

[DMM050905C85] Turnbull, J., Tiberia, E., Striano, P., Genton, P., Carpenter, S., Ackerley, C. A. and Minassian, B. A. (2016). Lafora disease. *Epileptic Disord.* 18, 38-62. 10.1684/epd.2016.0842 27702709 PMC5777303

[DMM050905C86] Upadhyay, M., Agarwal, S., Bhadauriya, P. and Ganesh, S. (2017). Loss of laforin or malin results in increased Drp1 level and concomitant mitochondrial fragmentation in Lafora disease mouse models. *Neurobiol. Dis.* 100, 39-51. 10.1016/j.nbd.2017.01.002 28063983

[DMM050905C87] Valles-Ortega, J., Duran, J., Garcia-Rocha, M., Bosch, C., Saez, I., Pujadas, L., Serafin, A., Cañas, X., Soriano, E., Delgado-García, J. M. et al. (2011). Neurodegeneration and functional impairments associated with glycogen synthase accumulation in a mouse model of Lafora disease. *EMBO Mol. Med.* 3, 667-681. 10.1002/emmm.201100174 21882344 PMC3377110

[DMM050905C88] Varea, O., Duran, J., Aguilera, M., Prats, N. and Guinovart, J. J. (2021). Suppression of glycogen synthesis as a treatment for Lafora disease: establishing the window of opportunity. *Neurobiol. Dis.* 147, 105173. 10.1016/j.nbd.2020.105173 33171226 PMC7736642

[DMM050905C89] Vernia, S., Rubio, T., Heredia, M., Rodríguez De Córdoba, S. and Sanz, P. (2009). Increased endoplasmic reticulum stress and decreased proteasomal function in lafora disease models lacking the phosphatase laforin. *PLoS One* 4, e5907. 10.1371/journal.pone.0005907 19529779 PMC2692001

[DMM050905C90] Worby, C. A., Gentry, M. S. and Dixon, J. E. (2006). Laforin, a dual specificity phosphatase that dephosphorylates complex carbohydrates. *J. Biol. Chem.* 281, 30412-30418. 10.1074/jbc.M606117200 16901901 PMC2774450

[DMM050905C91] Xu, R. and Salpeter, M. M. (1997). Acetylcholine receptors in innervated muscles of dystrophic mdx mice degrade as after denervation. *J. Neurosci.* 17, 8194-8200. 10.1523/JNEUROSCI.17-21-08194.1997 9334395 PMC6573762

[DMM050905C92] Yokoi, S., Austin, J., Witmer, F. and Sakai, M. (1968). Studies in myoclonus epilepsy (Lafora body form). I. Isolation and preliminary characterization of Lafora bodies in two cases. *Arch. Neurol.* 19, 15-33. 10.1001/archneur.1968.00480010033002 4175641

[DMM050905C93] Zeka, N., Zogaj, L., Gerguri, A., Bejiqi, R., Ratkoceri, R., Maloku, A., Mustafa, A., Shahini, L. and Maxharaj, J. (2022). Lafora disease: a case report. *J. Med. Case Rep.* 16, 360. 10.1186/s13256-022-03537-x 36192771 PMC9528140

[DMM050905C94] Zeng, L., Wang, Y., Baba, O., Zheng, P., Liu, Y. and Liu, Y. (2012). Laforin is required for the functional activation of malin in endoplasmic reticulum stress resistance in neuronal cells. *FEBS J.* 279, 2467-2478. 10.1111/j.1742-4658.2012.08627.x 22578008 PMC3407668

[DMM050905C95] Zimmern, V. and Minassian, B. (2024). Progressive myoclonus epilepsy: a scoping review of diagnostic, phenotypic and therapeutic advances. *Genes (Basel)* 15, 171. 10.3390/genes15020171 38397161 PMC10888128

[DMM050905C96] Zong, Y. and Jin, R. (2013). Structural mechanisms of the agrin-LRP4-MuSK signaling pathway in neuromuscular junction differentiation. *Cell. Mol. Life Sci.* 70, 3077-3088. 10.1007/s00018-012-1209-9 23178848 PMC4627850

[DMM050905C97] Zong, Y., Zhang, B., Gu, S., Lee, K., Zhou, J., Yao, G., Figueiredo, D., Perry, K., Mei, L. and Jin, R. (2012). Structural basis of agrin-LRP4-MuSK signaling. *Genes Dev.* 26, 247-258. 10.1101/gad.180885.111 22302937 PMC3278892

[DMM050905C98] Zutt, R., Drost, G., Vos, Y., Elting, W. J., Miedema, I., Tijssen, A. J. M., Brouwer, F. O. and De Jong, M., B. (2016). Unusual course of Lafora disease. *Epilepsia Open* 1, 1-4. 10.1002/epi4.12009 PMC5719837 29588937

