# Supplementary material for: Neuromuscular junction dysfunction in Lafora disease
Source: Dis Model Mech. 2024 Oct 14;17(10):dmm050905. doi: 10.1242/dmm.050905 (PMC11512103; doi:10.1242/dmm.050905)
Supplement: Supplementary information [file dmm-17-050905-s1.pdf]

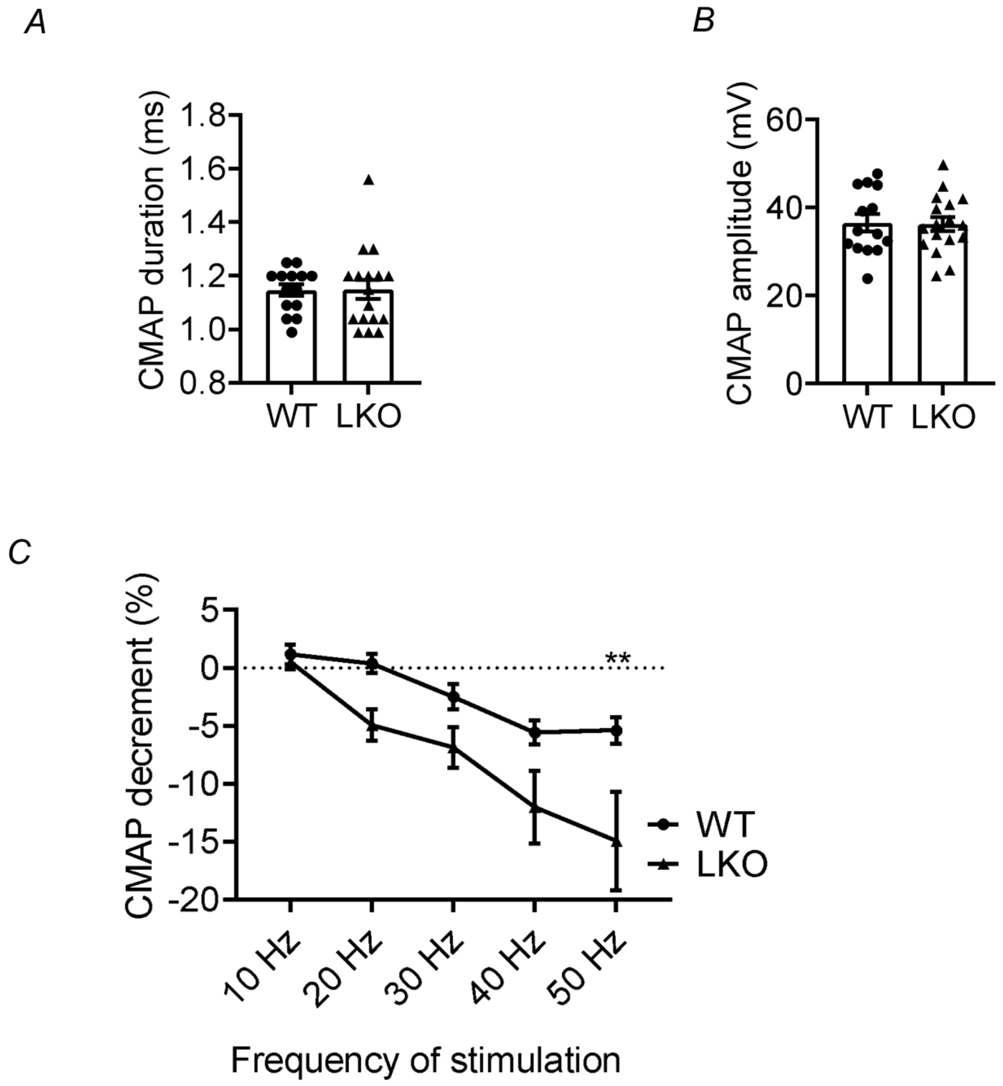

Fig. S1. *In vivo* electrophysiological assessment of NMJ transmission in Lafora disease mouse model. ***A-B:*** Bar diagram representing the CMAP duration (ms) and CMAP amplitude (mV) of 5-month-old wild-type (WT) and Laforin knock-out (LKO) mice (n = 14 [WT] and 17 [LKO]; unpaired two-tailed *t*-test, no significant difference found in CMAP duration (WT= 1.14 ms ± 0.02; LKO= 1.14 ms ± 0.03610; *p*-value=0. 9576) and CMAP amplitude (WT= 36.51 mV ± 1.95; LKO=36.24 mV ± 1.60; *p*-value=0.9139). ***C:*** Quantification of data representing the percentage CMAP decrement following RNS in 5-month-old LKO mice as compared to WT animals. Note the significantly increased CMAP decrement at 50 Hz frequency of stimulation in the LKO mouse model at 5 months. Each bar represents the mean ± SEM. (n = 14 [WT] and 17 [LKO]; two-way ANOVA with Sidak's multiple comparison test; **, $p<0.01$).

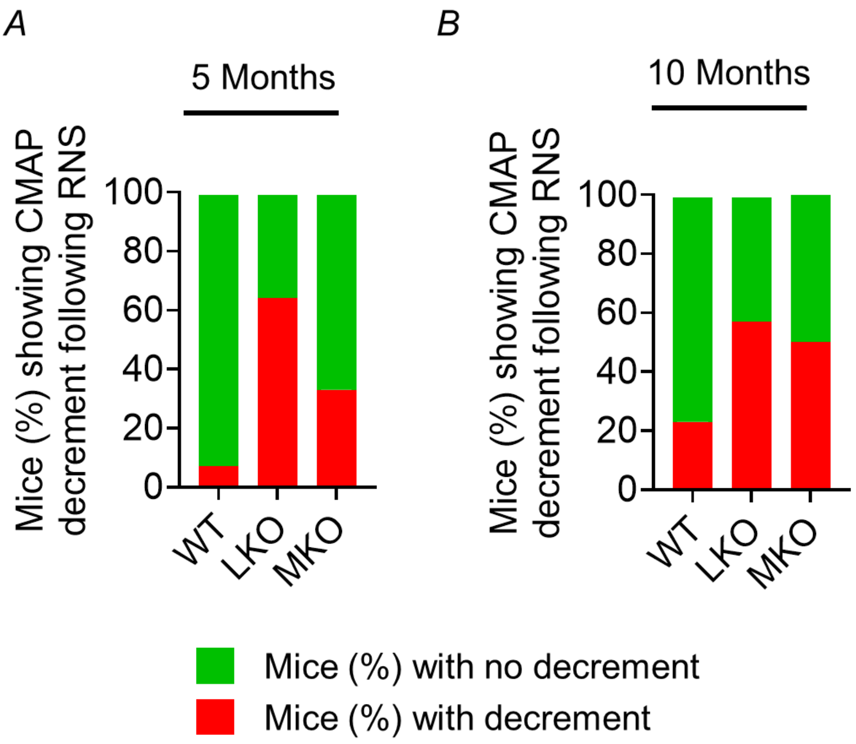

Fig. S2. NMJ transmission defects in the Lafora disease (LD) mouse model following repetitive nerve stimulation (RNS). Bar graph represents the proportion (%) of wild-type (WT), laforin-deficient (LKO), and malin-deficient (MKO) animals showing CMAP decrement at 50 Hz frequency for 5-month-old ***(A)*** and 10-month-old ***(B)*** animals (n = 14 [WT], 17 [LKO], and 12 [MKO] for the 5-month age group, and n= 17 [WT], 20 [LKO] and 18 [MKO] for the 10-month age group).

Disease Models & Mechanisms • Supplementary information

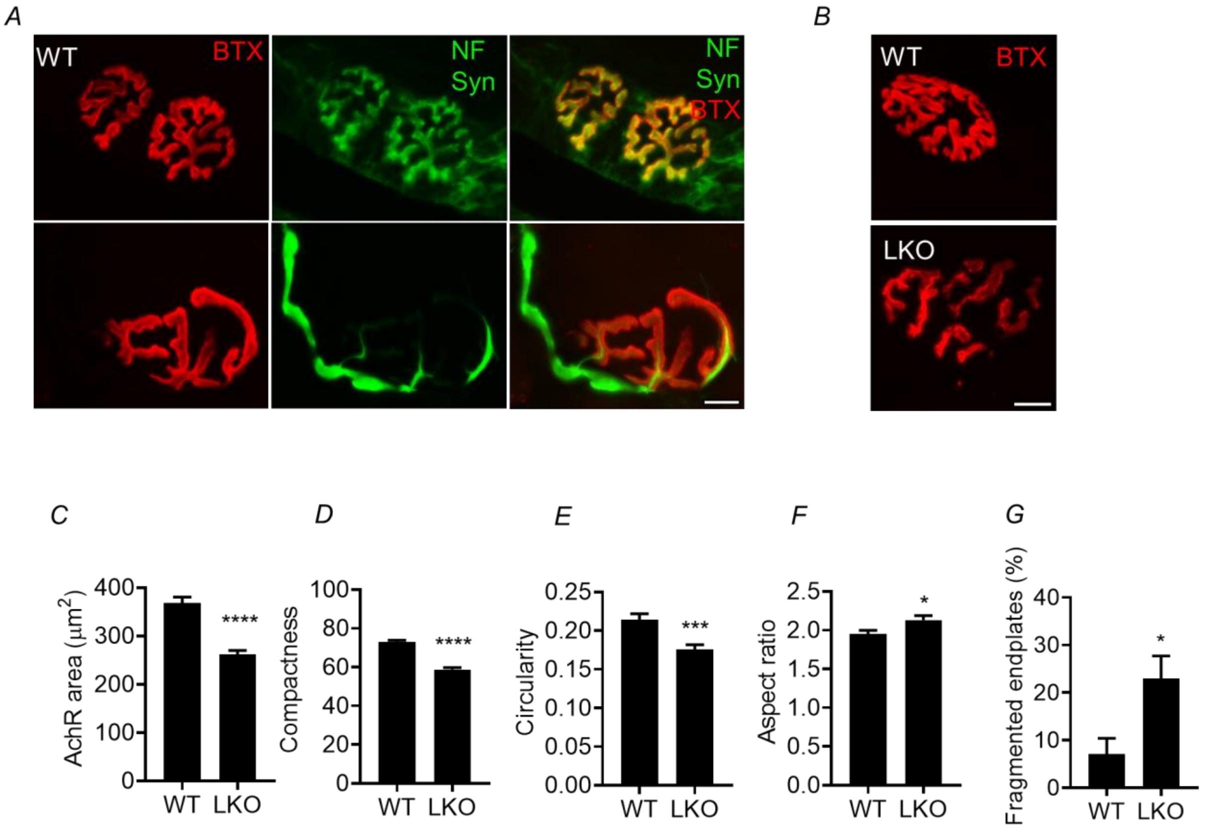

Fig. S3. Altered NMJ morphology in gastrocnemius muscle of Lafora disease mouse model. ***A:*** Representative immunofluorescence images showing NMJ morphology in 5-month-old wild-type (WT) and laforin-deficient (LKO) animals where postsynaptic acetylcholine receptors (AChRs) are stained with Alexa-594 conjugated α-Bungarotoxin (BTX; in red), the presynaptic nerve stained with anti-neurofilament (NF) antibody (in green), and presynaptic nerve terminals stained with synapsin-1 antibody (in green). Note the highly compact pretzel-shaped NMJ structure in WT animals. In contrast, dispersed and less complex NMJ morphology was observed in LKO mice. ***B:*** Representative immunofluorescence images showing a fragmented junction in LKO mice as opposed to an intact junction in WT mice at 5 months. ***C-F:*** Bar graphs show the quantification of NMJ morphological features as revealed by a significant reduction in postsynaptic AchR area ***(C)***, compactness ***(D)***, circularity ***(E)***, and an increase in aspect ratio ***(F)*** at 5-month-old animals. ***G:*** Bar graph shows the quantification of the relative percentage of fragmented endplates in LKO animals compared to WT animals in 5-month-old animals. Each bar represents the mean ± SEM. Here, 70-100 NMJs were analyzed for each animal and three animals were used for each genotype (WT and LKO) (unpaired two-tailed *t*-test; ****, $p<0.0001$; ***, $p<0.001$; *, $p<0.05$). Scale bar = 10 µm (***A*** and ***B***).

Disease Models & Mechanisms • Supplementary information

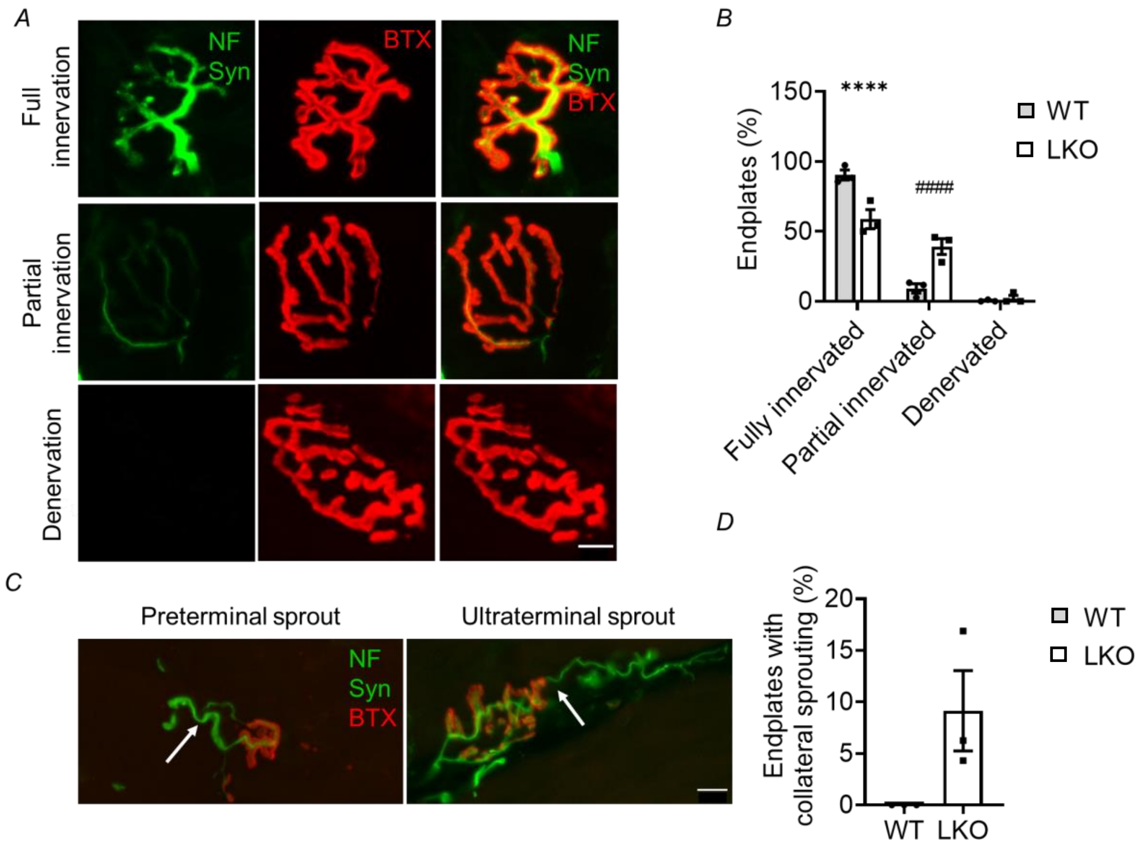

Fig. S4. NMJ innervation pattern and nerve collateral sprouting in gastrocnemius muscle of Lafora disease mouse model. ***A:*** Representative immunofluorescence images showing the NMJ innervation pattern categorized as full**,** partial, and denervated junctions. ***B:*** Bar graphs show the quantification of the relative percentage of endplates exhibiting full, partial, and denervated junctions in 5-month-old laforin-deficient (LKO) animals compared to their age-matched wild-type (WT) control animals. Each bar represents the mean ± SEM. Here, 70-100 NMJs were analyzed per animal, and three animals were used for each genotype (WT and LKO) (two-way ANOVA with Sidak's multiple comparison tests; $^{\#\#\#\#}$/*****p*<0.0001; an asterisk [*] is used to compare fully innervated junctions and hashtag [#] is used to compare the partially innervated junctions). ***C:*** Immunofluorescence images representing the phenomenon of collateral sprouting at a neuromuscular junction, which shows a rare example of preterminal sprouting (left) where the motor end plate is innervated by a newly formed thin axonal branch (indicated by white arrow) and an example of ultra terminal sprouting (right) where axon terminal develops the terminal branches and tries to innervate the nearby neighboring denervated junction (indicated by white arrow). ***D***: Bar graph shows the relative percentage of endplates with collateral sprouting in 5-month-old LKO animals as compared to their age-matched WT control animals. Each bar represents the mean ± SEM. Here, 70-100 NMJs were analyzed per animal and three animals were used for each genotype (unpaired two-tailed *t*-test; the difference was not statistically significant). Scale bar =10 µm (***A*** and ***C***).

Disease Models & Mechanisms • Supplementary information

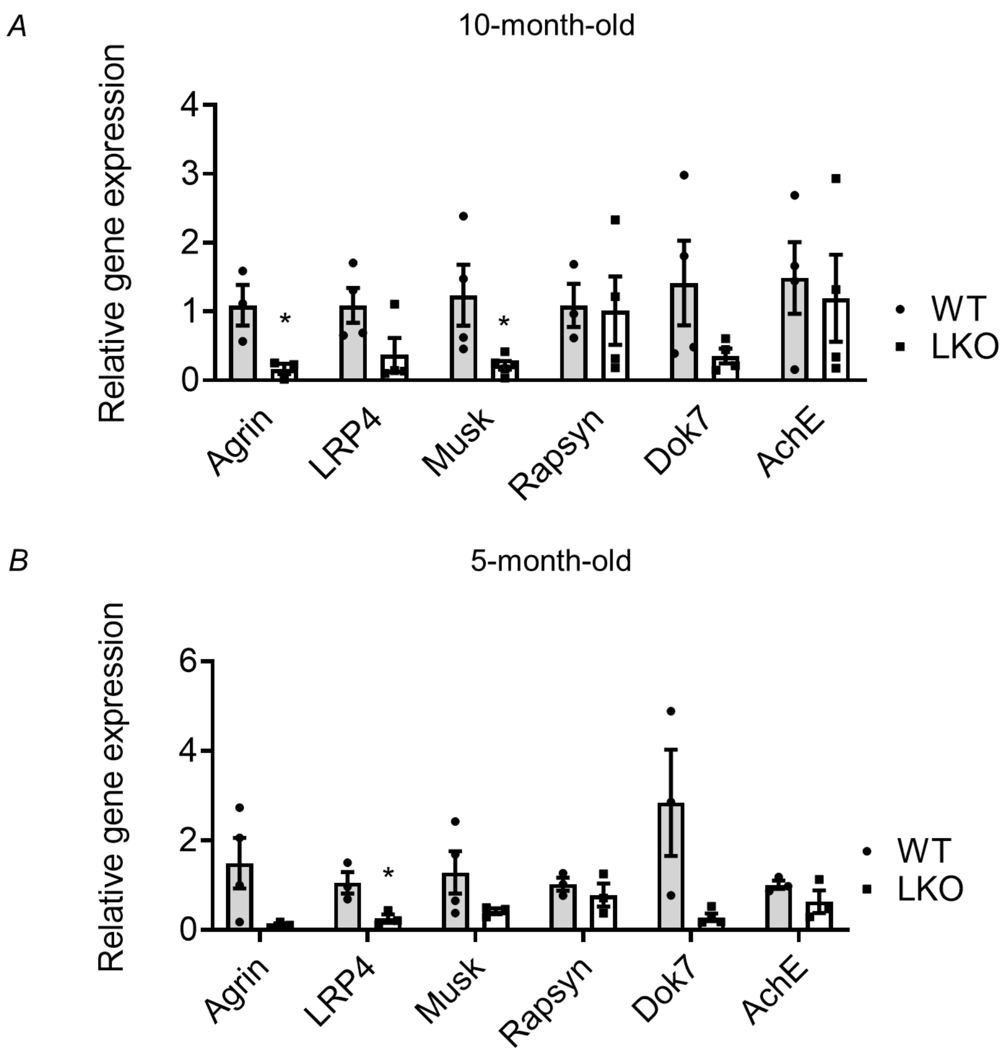

Fig. S5. Alteration in gene expression of NMJ-related transcripts by quantitative reverse transcriptase PCR. Bar graphs showing the relative gene expression normalized with *Gapdh* as an internal control in the 10-month-old (***A***) and 5-month-old (***B***) laforin-deficient (LKO) animals as compared to age-matched wild-type (WT) control animals. There is a significant downregulation in the expression of genes *Agrin* and *Musk* in 10-month-old LKO animals (*Agrin* [WT= 1.089 ± 0.2954, LKO = 0.1645 ± 0.07; two-tailed *t*-test, *p*-value = 0.0386], *Musk* [WT = 1.235 ± 0.4438, LKO = 0.2176 ± 0.06241, two-tailed *t-test*, *p*-value = 0.0371]) and *LRP4* gene in 5-month-old LKO mice (WT = 1.052 ± 0.2384, LKO = 0.2520 ± 0.09336; two-tailed *t-test*, *p*-value = 0.0353) as compared to their age-matched WT mice. s. Each bar represents the mean ± SEM. (n = 3-4 animals from each genotype [WT and LKO]; unpaired two-tailed *t*-tests; *, $p<0.05$).

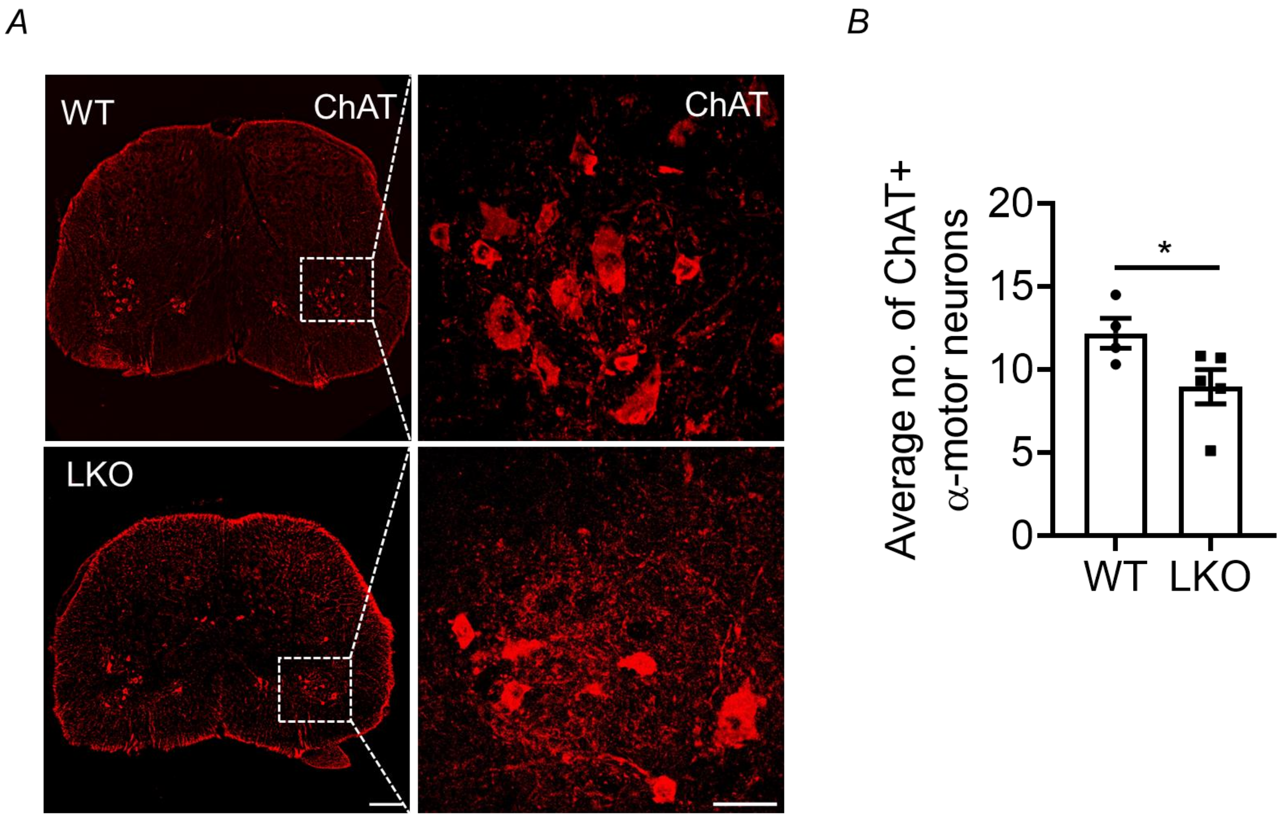

Fig. S6. Loss of ventral horn alpha motor neurons in the lumbar region of the spinal cord in 5-month-old Lafora (LD) mouse model. ***A:*** Representative immunofluorescence images of ChAT staining revealed the motor neurons in the ventral horn region of the lumbar spinal cord in the 5-month-old wild-type (WT) and laforin-deficient (LKO) animals. ***B:*** Bar graphs show the quantification of the average number of ChAT-positive α-motor neurons per side of the lumbar spinal cord in the ventral horn region in 5-month-old LKO animals as compared to their age-matched WT littermates. Each bar represents the mean ± SEM. (n = 4-5 animals for each genotype [WT and LKO]; unpaired two-tailed *t*-test; *, $p<0.05$). Scale bar = 200 µm (left) and 50 µm (right) in ***(A)*** .

Disease Models & Mechanisms • Supplementary information

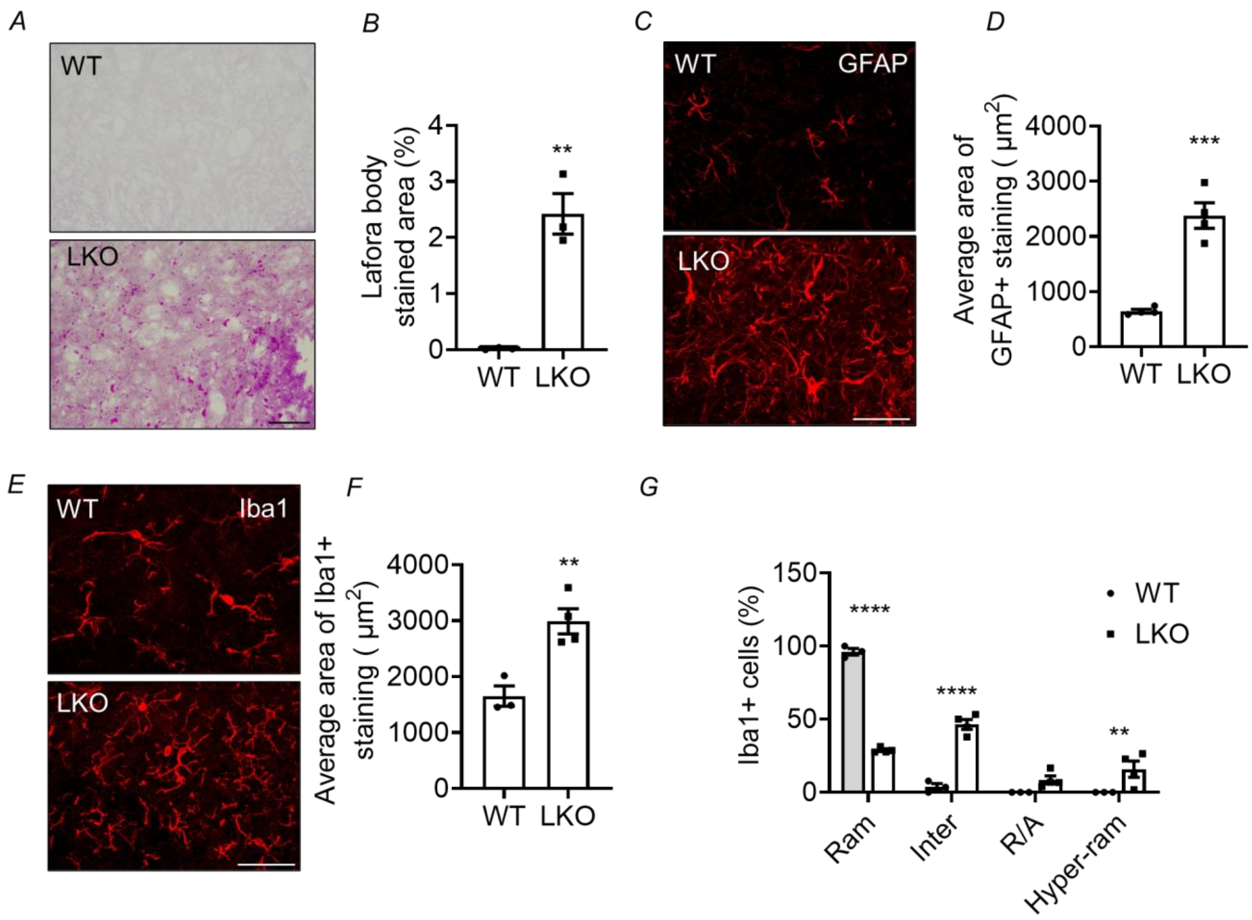

Fig. S7. Accumulation of Lafora bodies and increased gliosis in the lumbar region of the spinal cord in Lafora disease mouse model. ***A:*** Representative images showing the PAS-positive Lafora body accumulation in the ventral horn region of the spinal cord in the laforin-deficient (LKO) animal (wild-type, WT). ***B:*** Bar graph shows the percentage of Lafora body-positive stained area in the ventral horn region of the spinal cord in 5-month-old animals. Each bar represents the mean ± SEM. (n = 3 animals for each genotype [WT and LKO], unpaired two-tailed *t*-test; **, p<0.01). ***C:*** Representative images from spinal cord sections showing the distribution of GFAP+ astrocytes in the ventral horn region of WT and LKO animals. ***D:*** Bar graph compares the average area occupied by the GFAP+ cells in 5-month-old LKO animals with age-matched WT animals. Each bar represents the mean ± SEM. (n = 4 for each genotype [WT and LKO]; unpaired two-tailed *t*-test; ***, *p*<0.001). ***E:*** Representative images from spinal cord sections showing the distribution of Iba1+ microglial cells in the ventral horn region of WT and LKO animals. ***F:*** Bar graph compares the average stained area occupied by the Iba1+ cells in 5-month-old LKO animals with age-matched WT animals. Each bar represents the mean ± SEM. (n = 3 [WT] and 4 [LKO]; unpaired two-tailed *t*-test; **, *p*<0.01). ***G:*** Bar graph compares the relative percentage of microglial cells exhibiting the four different morphological phenotypes in LKO animals with WT control animals. Each bar represents the mean ± SEM. (n = 3 [WT] and 4 [LKO]; two-way ANOVA with Sidak's multiple comparison test; ****, *p*<0.0001, ***, *p*<0.001, **, *p*<0.01). Scale bar = 50 μm. (***A, C,*** and ***E***).

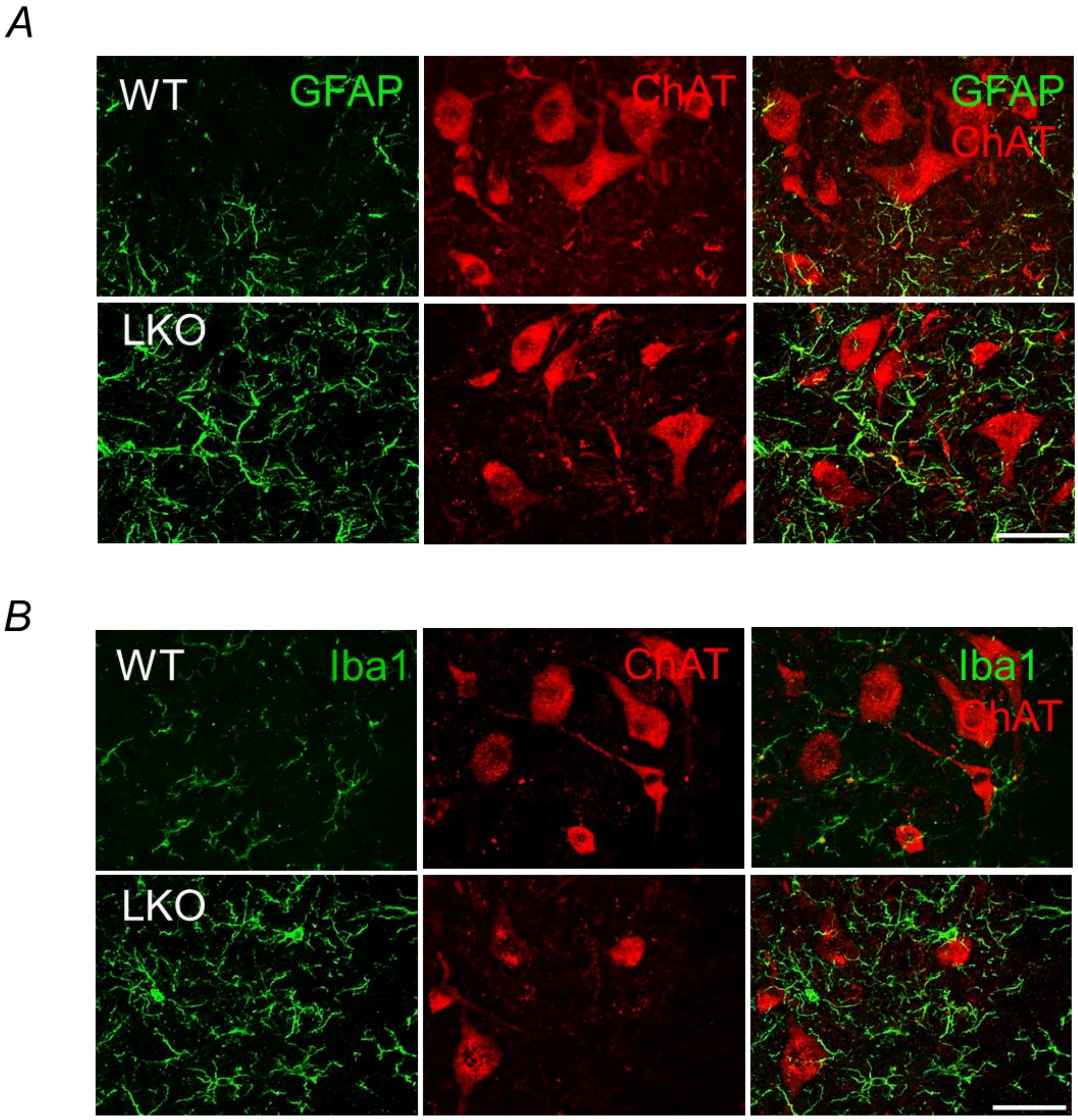

Fig. S8. Increased gliosis around the motor neurons in the lumbar region of the spinal cord in the LD mouse model. ***A:*** Representative images from spinal cord sections showing the double immunostaining in the ventral horn region of the spinal cord, which reveals the accumulation of astrocytes (GFAP, green) around the motor neurons (ChAT, red) in 10-month-old laforin-deficient (LKO) animals (wild-type, WT) ***B:*** Representative double immunostained images from spinal cord sections showing the distribution of Iba 1 positive cells (Iba1+, green) near the motor neurons (ChAT, red) in the 10-month-old LKO animals. Scale bar = 50 µm (***A*** and ***B***).

Disease Models & Mechanisms • Supplementary information

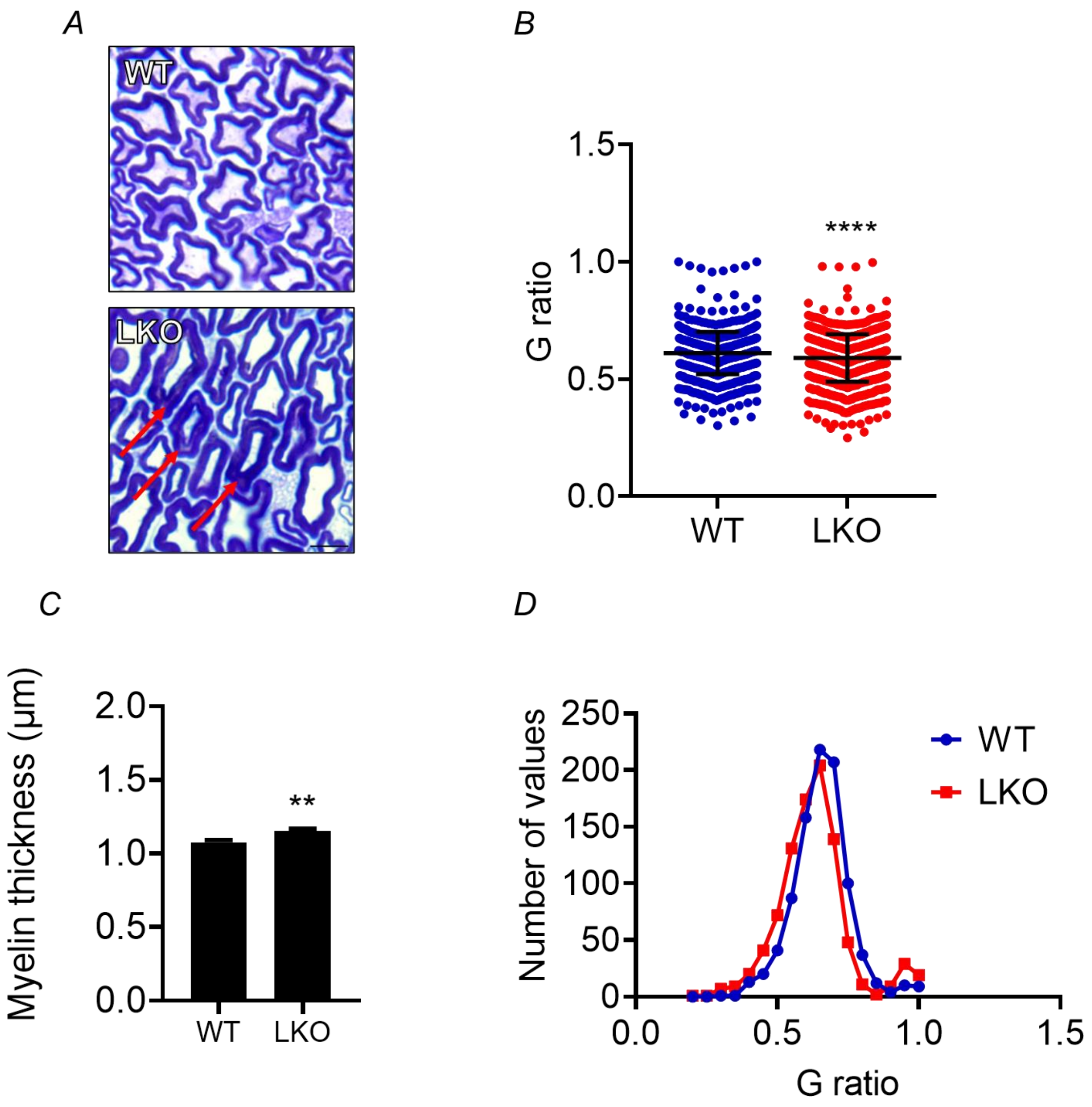

Fig. S9. The sciatic nerve morphology and myelination status are altered in the Lafora disease (LD) mice model. ***A:*** Representative images of semi-thin transverse sections of the sciatic nerve depicting nerve fiber morphology in 5-month-old wild-type (WT) and laforin-deficient (LKO) mice. Note the increased myelination in axons of LKO animals (indicated by red arrows). ***B-D:*** Bar graphs showing G ratio, myelin thickness, and relative distribution of G ratios across all myelinated nerve fibers in 5-month-old LKO animals as compared to WT animals. Axons of LKO animals show less G-ratio ***(B)*** when compared with WT animals which signifies more myelination ***(C)*** in the axons of LKO animals. For Figure B, each bar represents the mean ± SD, and for Figure C, each bar represents the mean ± SEM. Here, approximately 300 nerve fibers were analyzed from each animal, and three animals were used for each genotype (WT and LKO) (unpaired two-tailed *t*-test; ****, p<0.0001; **, p<0.01). Scale bar =10 μm.

Disease Models & Mechanisms • Supplementary information

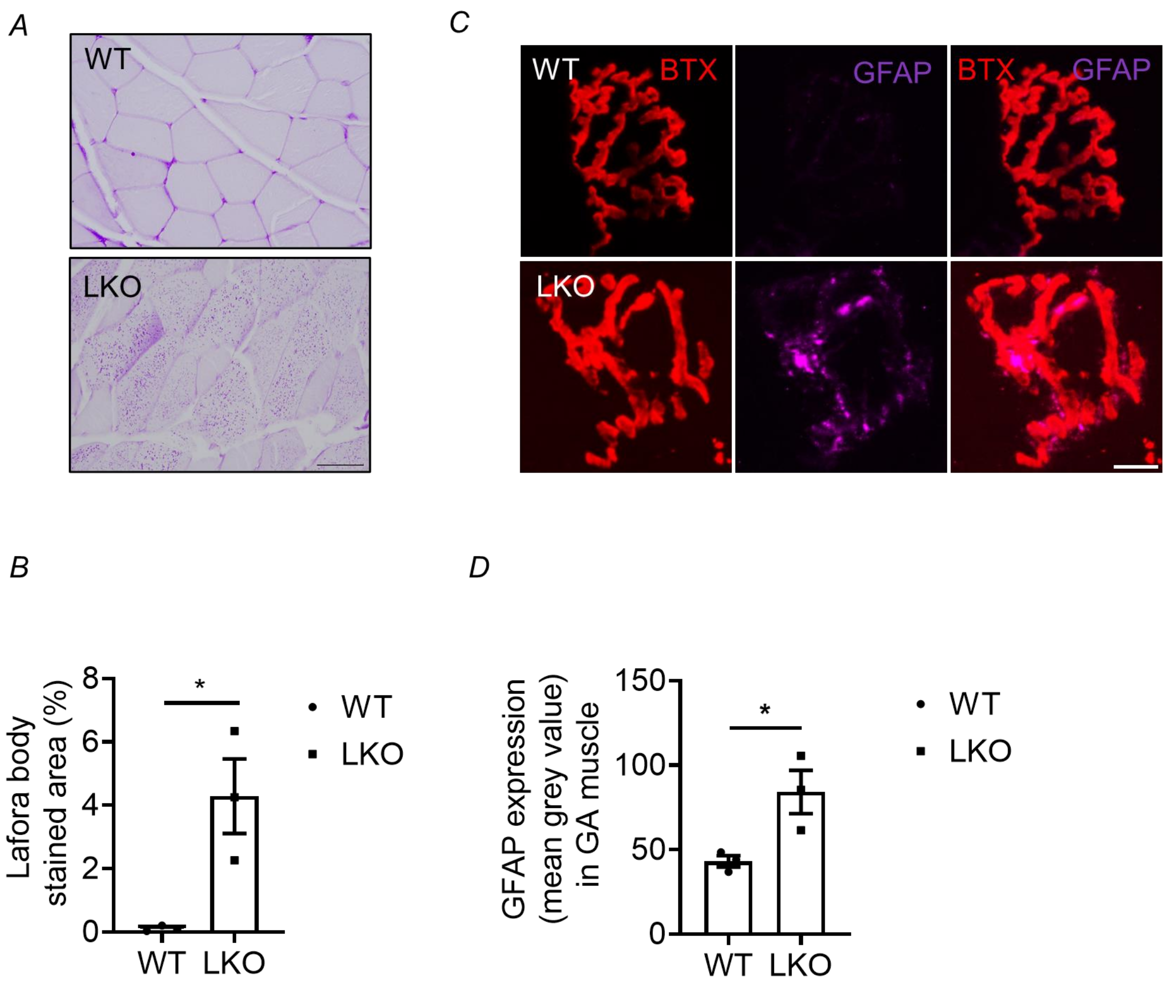

Fig. S10. Accumulation of Lafora bodies and increased GFAP expression in the gastrocnemius muscle of the Lafora disease mouse model. ***A:*** Representative images showing the PAS-positive Lafora body accumulation in the cross sections of the gastrocnemius muscle of the laforin-deficient (LKO) animal (wild-type, WT). ***B:*** Bar graph shows the percentage of Lafora body-stained area in the gastrocnemius muscle of 5-month-old LKO animals relative to age-matched WT animals. Each bar represents the mean ± SEM. (n = 3 animals for each genotype [WT and LKO]; unpaired two-tailed *t*-test; *, $p<0.05$). ***C:*** Representative images from gastrocnemius muscle sections showing the GFAP-BTX co-immunostaining in 5-month-old WT and LKO animals. Note the increased GFAP expression (GFAP, magenta) in the vicinity of the postsynaptic end plate region (BTX, red) in LKO animals. ***D:*** Bar graphs comparing the mean gray value of GFAP+ stained area within the motor endplate in the gastrocnemius muscle of 5-month-old LKO mice with age-matched WT control mice. Each bar represents the mean ± SEM. Here, 50-80 NMJs were analyzed per animal, and three animals were used for each genotype (WT and LKO); unpaired two-tailed *t*-test; *, $p<0.05$). Scale bar = 50 µm (***A***) and 10 µm (***C***).

Disease Models & Mechanisms • Supplementary information

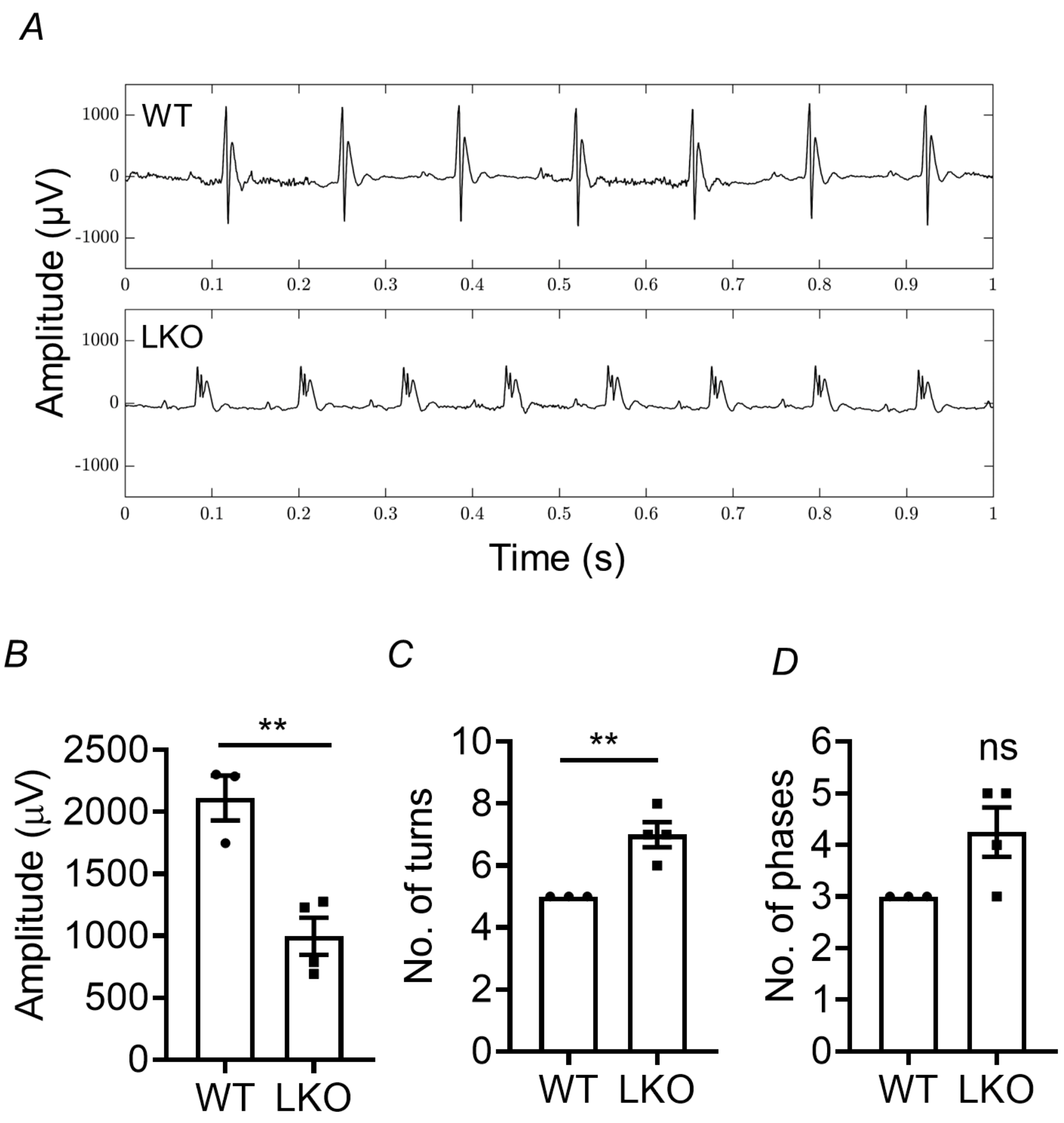

Fig. S11. Spontaneous electromyographic (EMG) assessment in Lafora disease mice model. ***A:*** Representative image shows the EMG traces from the WT (above) and LKO (lower) mouse model. Note the lower amplitude response and increased number of turns and phases in LKO mice. ***B-D:*** Bar diagram representing the amplitude (µV), number of turns, and number of phases in 10-month-old WT and LKO mice. Each bar represents the mean ± SEM. (n = 3-4 animals from each genotype (WT and LKO); unpaired two-tailed *t*-test, **, $p<0.01$; ns, $p = 0.07$).

Disease Models & Mechanisms • Supplementary information

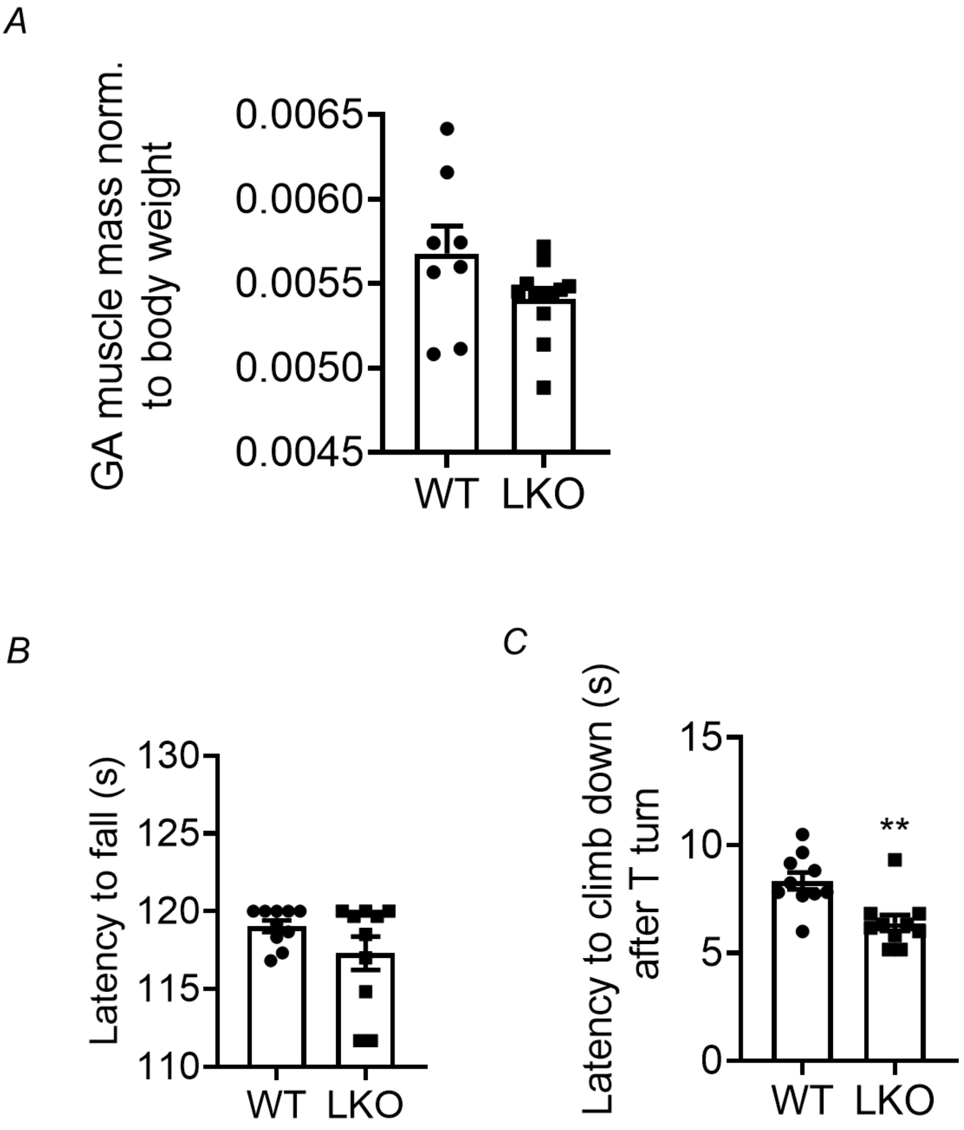

Fig. S12. Analysis of muscle mass, muscle strength, and motor performance in Lafora disease mouse model. ***A:*** Bar graph shows the gastrocnemius muscle mass normalized with body weight in 5-month-old laforin-deficient (LKO) animals as compared to wildtype (WT) control animals. Each bar represents the mean ± SEM. (n = 10 animals for each genotype [WT and LKO]; unpaired two-tailed *t*-tests; difference was not statistically significant) ***B-C:*** Animals were tested for inverted screen, and pole tests to evaluate muscle strength and motor coordination. No significant changes were observed between WT and LKO animals in the inverted screen test at 5 months *(B)*. However, 5-month-old LKO mice took significantly less time to reach their home cage as observed in the pole test. Each bar represents the mean ± SEM. (n = 10 animals for each genotype [WT and LKO]; unpaired two-tailed *t*-test, **, p<0.01.

Disease Models & Mechanisms • Supplementary information

Table S1. NMJ morphological evaluation of 10-month-old LKO mice

**Variables related to NMJ morphology**	**WT n=249 NMJs**	**LKO n=256 NMJs**	**Student *t*-test *p-value***
Acetylcholine receptor area (μm²)	537.8 μm2 ± 13.53	460.8 μm2 ± 11.45	p<0.0001
Compactness	75.56% ± 0.6481	67.64% ± 0.6875	p<0.0001
Circularity	0.3012 ± 0.009,	0.2599 ± 0.008	p<0.0015
Aspect ratio	1.854 ± 0.034	1.989 ± 0.047	p=0.0230
Fragmentation	=8.572% ± 3.39	29.67% ± 5.072,	p=0.0259

Data is expressed as mean ± SEM. Here, 70-100 NMJs were analyzed per animal, and three animals were used for each genotype (WT and LKO); unpaired two-tailed *t*-test (****, p<0.0001; **, p<0.01 *, $p < 0.05$).

Table S2. NMJ morphological evaluation of 5-month-old LKO mice

**Variables related to NMJ morphology**	**WT n=233 NMJs**	**LKO n=256 NMJs**	**Student *t*-test *p-value***
Acetylcholine receptor area (μm^2)	367.8 μm^2 ±12.64	262.3 μm^2 ±7.801	p<0.0001
Compactness	73.06%±0.779	58.69% ±1.002	p<0.0001
Circularity	0.214±0.007	0.178±0.006	p<0.0001
Aspect ratio	1.949±0.050	2.130±0.058	p=0.0208
Fragmentation	7.038±3.391	22.95±4.775	p=0.0532

Data is expressed as mean ± SEM. Here, 70-100 NMJs were analyzed per animal and three animals were used for each genotype (WT and LKO); unpaired two-tailed *t*-test (****, p<0.0001; *, $p < 0.05$).

Table S3. Nerve innervation pattern in 10-month-old WT and LKO mice

S. No.	Innervation pattern	Wild-type (WT) animals n= 249 NMJs	Laforin deficient (LKO) animals n= 256 NMJs	*p*-value
1	Fully innervated junctions	82.71% ± 4.45	34.838% ± 8.21	*p*-value<0.0001
2	Partially innervated junctions	16.87% ± 4.22	60.76% ± 5.73	*p*-value<0.0001
3	Denervated junctions	0.41% ± 0.41	4.399% ± 3.83	*p*-value= 0.5874 (ns)

Data is expressed as mean ± SEM. Here, 70-100 NMJs were analyzed per animal and three animals were used for each genotype (WT and LKO); Two-way ANOVA with Sidak's multiple comparison test; (****, $p<0.0001$; ns, not significant).

Disease Models & Mechanisms • Supplementary information

Table S4. Nerve innervation pattern in 5-month-old WT and LKO mice

S. No.	Innervation pattern	Wild-type (WT) animals n= 233 NMJs	Laforin deficient (LKO) animals n= 256 NMJs	*p*-value
1	Fully innervated junctions	90.37% ± 3.53	58.75% ± 6.75	*p*-value<0.0001
2	Partially innervated junctions	9.22% ± 3.27	39.16% ± 5.63	*p*-value<0.0001
3	Denervated junctions	0.40% ± 0.40	2.08% ± 2.08	*p*-value= 0.9013 (ns)

Data is expressed as mean ± SEM. Here, 70-100 NMJs were analyzed per animal and three animals were used for each genotype (WT and LKO); Two-way ANOVA with Sidak's multiple comparison test; (****, p<0.0001; ns, not significant).

Table S5. List of primers used for the RT-PCR

Gene	Forward primer 5’-3’	Reverse primer 5’-3’
Musk	5’-TGAGAACTGCCCCTTGGAACT-3’	5’-GGGTCTATCAGCAGGCAGCTT-3’
Agrin	5’-CCTCAACTTGGACACGAAGCT-3’	5’-AGGCCGATGCCCACAGA-3’
Dok7	5’-TCTCC CAGACCCGAGTTCTG-3’	5’-TCTAGCTGCAGGGCTTCCA-3’
LRP4	5’-GGACTGCACGTCAGCTATGC-3’	5’-CGCGATCACCAACAAAATCA-3’
Rapsyn	5’-ACGAGTGCGTGGAGGAGACT-3’	5’-TGTTCCTCTCCCCGATGGA-3’
AchE	5’-AAGGGCTGGGATATAATACGAC-3’	5’-CTTAGCCCAAGACATGCAGA-3’
Gapdh	5’-CGTGTTCCTACCCCCAATGT-3’	5’-TGTCATCATACTTGGCAGGTTTCT-3’
